# Cardiac implications of chicken wooden breast myopathy

**DOI:** 10.3389/fphys.2025.1547661

**Published:** 2025-03-05

**Authors:** Thea Parsberg Støle, Andreas Romaine, Thea Kleiberg, Vibeke Høst, Marianne Lunde, Almira Hasic, Tiril Aurora Lintvedt, Karen Wahlstrøm Sanden, Svein O. Kolset, Jens Petter Wold, Addolorata Pisconti, Sissel Beate Rønning, Cathrine Rein Carlson, Mona Elisabeth Pedersen

**Affiliations:** ^1^ Institute for Experimental Medical Research, Oslo University Hospital and University of Oslo, Oslo, Norway; ^2^ Raw Materials and Optimalization, Nofima As, Ås, Norway; ^3^ Department of Nutrition, Institute of Basic Medical Science, University of Oslo, Oslo, Norway; ^4^ Department of Biochemistry and Cell Biology, Stony Brook University, Stony Brook, NY, United States

**Keywords:** wooden breast, myopathy, heart, cardiac, broiler chicken

## Abstract

**Introduction:**

Wooden breast disease is a myopathy of the skeletal muscle in chickens of commercial breeding. Although the underlying pathophysiology remains unknown, we and others have previously shown that affected broilers display varying degrees of fibrosis, extracellular matrix (ECM) remodeling, inflammation, and alterations in various molecular signaling pathways. Other myopathy conditions, such as Duchenne muscular dystrophy, also affect the cardiac muscle and are associated with fibrosis and reduced cardiac function. To determine potential cardiac implications of wooden breast disease and identify whether molecular and fibrotic changes were similar to what we have previously found in the breast, we have investigated the hearts of commercial Ross 308 broilers.

**Methods:**

Hearts from male Ross 308 broiler chickens from mildly and severely wooden breast-affected chickens categorized in previous studies were analyzed. Ventricles from the hearts were analyzed by immunoblotting, real-time qPCR, near-infrared spectroscopy, Raman spectroscopy, and Masson`s trichrome histology. RNA sequencing was also conducted to identify the molecular footprint of the mildly and severely wooden breast-affected chickens.

**Results:**

Compared to mildly affected chickens, the severely wooden breast-affected chickens did not show an increase in heart weight, water-binding capacity, or macronutrient composition. The hearts did also not display any differences in fibrosis development, extracellular matrix gene expression, or typical cardiac and inflammatory markers. The severely affected chickens did, however, show a reduction in protein levels of biglycan and fibromodulin, as well as alterations in matrix metalloproteinase 2, Wnt ligands, mTOR signaling, heat shock protein 70, and muscle LIM protein. Functional enrichment analysis of RNA sequencing also suggested a different molecular footprint of biological processes and pathways between the two groups.

**Conclusion:**

Hearts from wooden breast-affected chickens did not display the same fibrotic alterations as those previously found in the breast. Despite few alterations detected in the markers and signaling molecules tested, RNA sequencing indicated a different molecular footprint in the hearts of severely compared to mildly wooden breast-affected chickens.

## 1 Introduction

Wooden breast disease (WB) is a myopathy of the skeletal breast muscle (*Pectoralis major*) in fast-grown broilers (Sihvo et al., 2014; Mudalal et al., 2015), with incidences reported as high as 96.1% (Tijare et al., 2016). Macroscopically, the condition is characterized by a rigid hardening of the tissue, especially at the caudal end of the breast, leading to a reduction in the physical and chemical quality of the meat (de Almeida Assunção et al., 2020; Xing et al., 2020). Due to the growing demand for poultry meat and an increase in the incidence of WB, the condition leads to huge economic losses in the industry (Kuttappan et al., 2016; Zanetti et al., 2018).

While the underlying pathophysiology of the condition remains to be fully understood, it is thought to be induced by the genetic selection of faster-growing broilers, leading to the development of spontaneous myopathies (Petracci et al., 2015; Forseth et al., 2023). Pairing to the name, WB-affected breasts have been found to display fibrosis and immune cell infiltration, resulting in tough and hardened tissue (Sihvo et al., 2014). RNA sequencing of WB-affected breasts has shown an upregulation of genes involved in intracellular calcium regulation, hypoxia, oxidative stress, fiber-type switching, and cellular repair (Mutryn et al., 2015). Our previous molecular studies also suggests alterations in signaling pathways such as mitogen-activated protein kinases (MAPK), Akt and Wnt, and matrix metalloproteinases (MMPs) and transmembrane syndecan shedding in the affected breasts (Pejšková et al., 2023; Pejšková et al., 2024).

While WB is primarily recognized as a myopathy of the skeletal muscle in broilers, skeletal and cardiac muscle share similarities. Myocytes of the skeletal and cardiac muscles both originate from the mesoderm during embryonic development (Costamagna et al., 2014). Like skeletal myocytes, cardiomyocytes contain sarcomeres which facilitate their contractility. However, while the skeletal muscle is under voluntary control, the cardiac muscle is not. A synchronized and coordinated contraction and relaxation cycle of the myocardium is initiated by electrical simulation from the sinus node, allowing the heart to supply the body’s organs with the appropriate oxygen and nutrient supply it requires (Stephenson et al., 2016). As such, an increase and accumulation of extracellular matrix (ECM) proteins leads to the stiffening of the heart, associated with a decline in function, ultimately leading to progressive heart failure (Czubryt and Hale, 2021).

Whether WB disease of the broiler breast muscle is associated with changes also in the cardiac muscle has, to our knowledge, not been investigated before. However, similar molecular alterations, such as an increase in syndecan levels and shedding, are observed in the WB-affected breast, and also in the dysfunctional heart (Strand et al., 2015; Herum et al., 2020; Pejšková et al., 2023). Additionally, other myopathies, such as Duchenne muscular dystrophy and inflammatory myopathies, exhibit a correlation between skeletal muscle and cardiac dysfunction (Posner et al., 2016; Prisco et al., 2020). We therefore hypothesized that the heart may be affected by WB and that similar mechanisms of inflammation and fibrotic development may be present. In this study, we investigated the cardiac implications associated with WB disease of the chicken to identify potential alterations in cardiac structure and fibrosis. We also analyzed molecular changes in the ECM, signaling pathways, cardiac and inflammatory markers, and syndecan gene and protein expression.

## 2 Materials and methods

### 2.1 Animal sampling and classification of the chicken hearts

Male Ross 308 broiler chickens (*Gallus gallus*) were fed a diet of wheat/maize pellets *ad libitum*, from the age of 10 days. Chickens were housed in 2.4 × 0.95-m pens covered in wood shavings with 6:18 h light:dark cycles with gradually reduced temperatures of 28 to 21^°^C. Upon slaughter at 36 days post-hatching, the atria were removed, and the ventricles were snap-frozen in liquid nitrogen. The hearts were from the same chickens we have previously analyzed the breasts from and were grouped (mild and severe) based on the previous WB classification of the chicken breasts (Pejšková et al., 2023). The classification was based on manual palpitation of the breast, followed by histological analysis where the severe group showed marked fibrosis and collagen infiltration in the breast, whereas the mild group showed few signs (Pejšková et al., 2023). Since all chickens of the Ross308 breed seem to display some signs of skeletal muscle myopathy (Pejšková et al., 2023; Pejšková et al., 2024), we have compared cardiac samples from mildly and severely WB-affected chickens.

### 2.2 Immunoblotting and blocking peptides

Tissue from the ventricles of mild and severely affected chickens was homogenized with TissueLyser (#85300, Qiagen Nordic, Venlo, Netherlands) in ice cold lysis buffer (20 mM Hepes (pH7.5), 150 mM NaCl, 1 mM EDTA, and 0.5% Triton-X100), supplemented with complete EDTA-free protease inhibitor cocktail (#5056489001, Roche Applied Science, Merck, Darmstadt, Germany) and PhosSTOP (#4906837001, Roche Applied Science, Merck). Homogenates were centrifuged (19 cm rotor diameter) at 14,000 rcf for 10 min at 4^°^C before supernatants were stored at −80^°^C. Protein concentrations were determined by using a Micro BCA protein assay kit (#PIER23235, Thermo Fisher Scientific, MA, United States). 40 μg protein was loaded per lane on 26- or 18-well 4%–15% Criterion TGX precast gels (#5671085 and #5671084, Bio-Rad, CA, United States) before transferring onto PVDF membranes (#1704157, Bio-Rad, or #0301004001, Merck) using a Trans-Blot Turbo system (Bio-Rad). Membranes were blocked in 1 × casein (#11836170001, Merck) or 5% BSA (#805090, Norsk Labex, Høvik, Norway) for 1 h at room temperature and thereafter incubated with primary antibodies overnight at 4^°^C. Following incubation, membranes were washed in TBS-T [Tris-buffered saline with 1% Tween-20 (#1610781, Bio-Rad)] for 20 min, followed by three 10-min washes. HRP-conjugated secondary antibodies were then added for 1 h at room temperature before the membranes were washed for 20 min, followed by four 5-min washes in TBS-T. Blots were developed with ECL prime (#RPN2236, Cytiva, MA, United States) and signal was detected using Azure 600 Western blot imaging system (Azure Biosciences, CA, United States). Membranes were stripped for 5–10 min (#PIER21603, Thermo Scientific, CA, United States) before reprobing. Equal loading was confirmed with GAPDH or Revert 700 protein staining (#926-11021, LI-COR Biosciences, NE, United States). A list of primary antibodies, blocking conditions, dilutions, molecular weight detected by immunoblotting, and the Uniprot protein IDs are listed in Table 1.

**TABLE 1:** Primary antibodies used for immunoblotting.

Protein name:	Uniprot:	Western blot kDa:	Blocking condition:	Antibody:	Dilution:	Supplier:
Wnt3a	Q2LMP1 WNT3A_CHICK (352aa)	45–55 kDa	1 × Casein	ab28472	1:1,000	Abcam
Wnt7a	Q9DEB8 Q9DEB8_CHICK (349a)	40–50 kDa	5% BSA	ab100792	1:1,000	Abcam
Biglycan	Unidentifiable (369aa)	40 kDa	1 × Casein	sc-27936	1:500	SantaCruz
Fibromodulin	P51887 FMOD_CHICK (380aa)	40 kDa	1 × Casein	sc-33772	1:500	SantaCruz
pSer2448-mTOR	F1NUX4 F1NUX4_CHICK (2521aa)	260 kDa	5% BSA	#2971	1:1,000	Cell Signaling
mTOR				#2983		
HSP70	P08106 HSP70_CHICK (634aa)	70 kDa	1 × Casein	sc-373867	1:500	SantaCruz
Wnt4	P49337 Wnt4_CHICK (351aa)	35 kDa	1 × Casein	ab91226	1:1,000	Abcam
Decorin	P28675 PGS2_CHICK (357aa)	50–75 kDa	1 × Casein	AF1060	1:500	R&D systems
LOX	Q05063 LYOX_CHICK (420aa)	42 kDa	1 × Casein	sc-32409	1:500	SantaCruz
MLP	F1NWZ2 F1NWZ2_CHICK (194aa)	20 kDa	1 × Casein	sc-166930	1:1,000	SantaCruz
pThr180Tyr182-p38 MAPK	A0A1D5PIQ5 A0A1D5PIQ5_CHICK (360aa)	40 kDa	5% BSA	#9211	1:1,000	Cell Signaling
p38 MAPK				ab31828	1:1,000	Abcam
pSer473-Akt	057513 057513_CHICK (480aa)	55 kDa	5% BSA	#9271	1:500	Cell Signaling
Akt				#9272	1:500	Cell Signaling
Syndecan-1	F1NV24 F1NV24_CHICK (308aa)	10–250 kDa	1 × Casein	Custom made (Pejšková et al., 2023)	1:1,000	Genscript
Syndecan-2	Q8JIY0 Q8JIY0_CHICK (201aa)	10–75 kDa	1 × Casein	Custom made (Pejšková et al., 2023)	1:1,000	Genscript
Syndecan-3	P26261 SDC3_CHICK (405aa)	10–150 kDa	1 × Casein	Custom made (Pejšková et al., 2023)	1:1,000	Genscript
Syndecan-4	P49416 SDC4-CHICK (197aa)	15–150 kDa	1 × Casein	Custom made (Pejšková et al., 2023)	1:1,000	Genscript
MMP9	A0A8V0YXL9 A0A8V0YXL9_CHICK (688aa)	80 kDa	1 × Casein	NBP1-57940	1:1,500	Novus Biologicals
MMP2	Q90611 MMP2_CHICK (663aa)	66–72 kDa	1 × Casein	ab181286	1:1,500	Abcam
Cardiac troponin T	P02642 TNNT2_CHICK (302aa)	40 kDa	1 × Casein	MA5-12960	1:500	Invitrogen
GAPDH	P00356 G3P_CHICK (333aa)	35 kDa	1 × Casein	sc-47724	1:500	SantaCruz

For the four syndecan proteins, blocking peptides against the antibody epitopes were used to analyze the specificity of bands detected with immunoblotting. Here, custom-made blocking peptides (Genscript, NJ, United States) against chicken syndecan-1-4 were pre-incubated with the respective antibodies before immunoblotting of the membranes. The syndecan antibodies and their corresponding blocking peptides were previously used and epitope mapped in Pejšková et al. (2023).

#### 2.2.1 Blocking peptides

Syndecan-1: NGGYQKPHKQE.

Syndecan-2: RKPSSAAYQKAPTK.

Syndecan-3: KQANVTYQKPDKQE.

Syndecan-4: DLGKKPIYKKAPTN.

#### 2.2.2 Antibodies

Secondary HRP-conjugated antibodies were anti-rabbit IgG HRP (N934V, Cytiva, MA, United States) and anti-mouse IgG HRP (NA931V, Cytiva).

### 2.3 Real-time quantitative PCR

Total RNA was extracted according to the manufacturer’s instructions using the RNeasy Midi Kit (#75144, Qiagen, Germany) and lysis with Precellys Lysing Kit (#P000911-LYSKO-A.0, Bertin technologies, Montigny-le-bretonneux, lle-de-France, France); 6,000 rpm 4 × 20 s intervals. cDNA was generated from 2 µg RNA using Taqman Reverse Transcription Reagents (#N8080234, Thermo Fisher Scientific, MA, United States) in a 40 µL reaction volume with random hexamers according to the manufacturer’s protocol. RT-qPCR analysis was carried out using TaqMan Gene expression Master Mix (#4369510, Life Technologies, Thermo Fisher Scientific) and the QuantStudio5 (Applied Biosystems, Foster City, CA, United States) PCR System. The amplification protocol was initiated at 50^°^C for 2 min, followed by denaturation at 95^°^C for 10 min, then 40 cycles of denaturation at 95^°^C for 15 s followed by annealing of TaqMan probes and amplification at 60°C for 1 min. RT-qPCR analyses were performed with 3 technical replicates from each sample. The relative gene expression (RQ) was calculated by the comparative 2^−ΔCt^ method (Schmittgen and Livak, 2008; Bustin et al., 2009). Normalization was performed against the eukaryotic translation elongation factor 2 (*EEF2*) reference gene for each sample, and subsequently related to the average gene expression of the mild samples for each gene analyzed. All TaqMan^®^ primers and probes are listed in Table 2.

**TABLE 2:** Gene target and TaqMan primer assays.

TaqMan^®^ probes	Gene name	Assay ID
Actin Alpha 2	*ACTA2*	Gg03352404_m1
Biglycan	*BGN*	Gg07177841_m1
Collagen type 1	*COL1A1*	Gg07167955_g1
Collagen type 3	*COL3A1*	Gg03325764_m1
Decorin	*DCN*	Gg03355063_m1
Desmin	*DES*	Gg03330588_m1
Eukaryotic translation elongation factor 2	*EEF2*	Gg03339740_m1
Interleukine 1 beta	*IL-1B*	Gg03347157_g1
Lysyl Oxidase	*LOX*	Gg03340182_m1
Lumican	*LUM*	Gg03325844_m1
Matrix metalloproteinase-2	*MMP2*	Gg03365277_m1
Matrix metalloproteinase-9	*MMP9*	Gg03338324_g1
Platelet-derived growth factor receptor beta	*PDGFRB*	Gg07165531_s1
Syndecan-1	*SDC1*	Gg07175697_s1
Syndecan-2	*SDC2*	Gg03345645_m1
Syndecan-3	*SDC3*	Gg03339851_m1
Syndecan-4	*SDC4*	Gg03370419_m1
Transforming growth factor beta 1	*TGFB1*	Gg07156069_g1
Tissue inhibitor of metalloproteinases 2	*TIMP2*	Gg07157666_m1
Toll like receptor 4	*TLR4*	Gg03354643_m1
Tropomyosin beta-chain	*TPM2*	Gg03815778_s1
Troponin T2 cardiac type	*TNNT2*	Gg03371505_m1
Tubulin alfa-1	*TUBA1A*	Gg07162375_m1
Tubulin beta-1	*TUBB1*	Gg03371486_g1

### 2.4 Near-infrared spectroscopy

Near-infrared spectroscopy (NIRS) is an established technique for the rapid detection of WB syndrome in chicken breasts, a method which is also developed for industrial sorting (Wold et al., 2017; Wold et al., 2019). The lower content of protein and the more loosely bound water in the affected muscle can easily be quantified by NIRS. Therefore, it was interesting to evaluate if NIRS can distinguish between heart muscles from mild and severely affected chickens. The handheld instrument MicroNIR PAT-U (VIAVI Solutions Inc., AZ, United States) is based on a 128-pixel InGaAs photodiode array and a linear filter. It collects spectra in the wavelength region 908–1,676 nm. Two LEDs are used for illumination, and an approx. circular area of D = 10 mm was probed with the system. Spectral collections were done in physical contact with the heart sections. Each measurement integrated spectra over 1 s. In the analysis, we used the absorption spectra and spectra normalized by standard normal variate (Barnes et al., 1989) to remove the main effect of the light scattering on the spectra.

### 2.5 Raman spectroscopy

Raman spectroscopy was used to get an indication of collagen accumulation in the hearts. A MarqMetrix All-in-One (AIO) Raman system covering a Raman shift range of 100–3,250 cm^−1^ was employed. The system was equipped with a 785 nm laser operating at 450 mW power and the sampling optic was a wide area illumination (D = 3 mm) Proximal BallProbe HV stand-off Raman probe (MarqMetrix Inc., WA, United States) operating at a 10 cm working distance. The left ventricle (LV) was scanned under illumination for 25 s three times, using the average spectrum for further analyses. Spectra were baseline corrected, meaning that the broad band signal associated with autofluorescence was removed to retain Raman signals only. The ability of Raman spectroscopy to characterize different types of collagen structures has been demonstrated in many studies (Rygula et al., 2013; Martinez et al., 2019). More recent work (Monago-Maraña et al., 2021; Lintvedt et al., 2024) has also shown that the quantification of hydroxyproline, as a proxy for collagen, in heterogeneous poultry materials is possible, using appropriate probes with larger laser spot sizes. These studies pointed at unique spectral fingerprints for collagen compared to general proteins by the peak intensity ratios at Raman shifts 827/854 cm^−1^, 919/936 cm^−1^, and 1,657/1,678 cm^−1^. Therefore, these ratios were used as collagen markers in this study.

### 2.6 Masson’s trichrome histology and quantification

The LV was excised, washed in ice-cold PBS, segmented, and snap-frozen in liquid nitrogen. LV segments were embedded in Tissue-Tek®O.C.T.™ compound (Sakura Company, CA, United States) and sectioned into 7 μm using a Cryostar NX70 Cryostat (Thermo Scientific, MA, United States). To quantify collagen, sections were stained with Masson’s trichrome stain according to the manufacturer’s protocol (Polysciences, PA, United States) with the following modifications: slides were cleared with Histoclear II (National Diagnostics, GA, United States) and mounted using VectaMount Express (Vector Laboratories, CA, United States). Images were captured using a ×20 objective on an AxioScan Z1 (Carl Zeiss, Germany), compositing tiles of the whole LV segment. ImageJ (NIH) was used to remove artifacts and residual pericardial tissue if present, then processed images were exported to Zen 2 (Carl Zeiss, Germany) where colour thresholding was used to measure positive pixel area as a proportion of total pixel area. Analysis was conducted by a researcher blinded for phenotype, and entire LV segments were captured and analysed to avoid selection biases.

### 2.7 RNA sequencing

RNA was extracted using RNeasy Mini Kit and sent to Novogene for library preparation and sequencing. At Novogene, messenger RNA was purified from total RNA using poly-T oligo-attached magnetic beads. After fragmentation, the first strand cDNA was synthesized using random hexamer primers followed by the second strand cDNA synthesis. The library was ready after the end repair, A-tailing, adapter ligation, size selection, amplification, and purification. The library was checked with Qubit and real-time PCR for quantification and bioanalyzer for size distribution detection. Quantified libraries were pooled and sequenced on an Illumina a Novoseq6000 instrument. Sequencing QC was performed with FastQC v0.12.1 (Andrews, 2010), Trim Galore v0.6.7 (Krueger et al., 2021) [and Cutadapt v3.4 (Martin, 2011)]. Reads were mapped to the GRCg7b version of the *Gallus Gallus* reference genome using STAR v2.7.9a (Dobin et al., 2013). Alignments were converted to BAM format and sorted using samtools v1.17 (Li et al., 2009). Transcript expression was then quantified with Salmon v1.10.1 (Patro et al., 2017), and converted to gene-level counts with Tximport v1.12.0 (Soneson et al., 2015). Differential expression analysis comparing the severe and mild groups was next performed using the nf-core differential abundance pipeline v1.4.0 (WackerO et al., 2023), with read count normalization and statistical analysis performed with DESeq2 v1.34.0 (Love et al., 2014). The results of the differential expression analysis were visualized using the Enhanced Volcano R package v1.20.0.

Gene set enrichment analysis (GSEA) was performed with the ClusterProfiler R package v4.10.1 (Yu et al., 2012; Wu et al., 2021) using the fgsea algorithm (Korotkevich et al., 2016). Log2 fold-change scores were used to rank genes, and the results were visualized with the Enrichplot package v1.22.0 (Wu et al., 2021). Network analysis to find genes with correlated expression levels was performed with WGCNA v.1.72.5 (Langfelder and Horvath, 2008) using normalized and variance-stabilized read counts and a soft-thresholding power value of 10. The genes present in selected modules were then subject to functional overrepresentation analysis (ORA) using ClusterProfiler v4.10.1. For the ORA, the background gene list was defined as the set of genes used in the differential expression analysis that also possessed relevant functional annotations.

### 2.8 Statistics analysis

Immunoblots are displayed as mean ± SEM and qPCR data are presented as the fold change average relative to the mean of the mildly affected samples. qPCR data of mild and severely affected groups were compared using Welch’s t-test due to our observation of a larger spread in the breasts of the same severely affected chickens (Pejšková et al., 2023). Mann-Whitney *U*-tests or student’s *t*-tests were used for quantified immunoblots due to non-normal or normal distribution, respectively, analyzed by Shapiro-Wilk testing.

Principal component analysis was used to decompose the NIR spectra into a few principal components to detect the grouping of samples and characterize potential spectral differences between these.

## 3 Results

### 3.1 Characterization of hearts from mild and severely wooden breast-affected chickens

**FIGURE 1:**
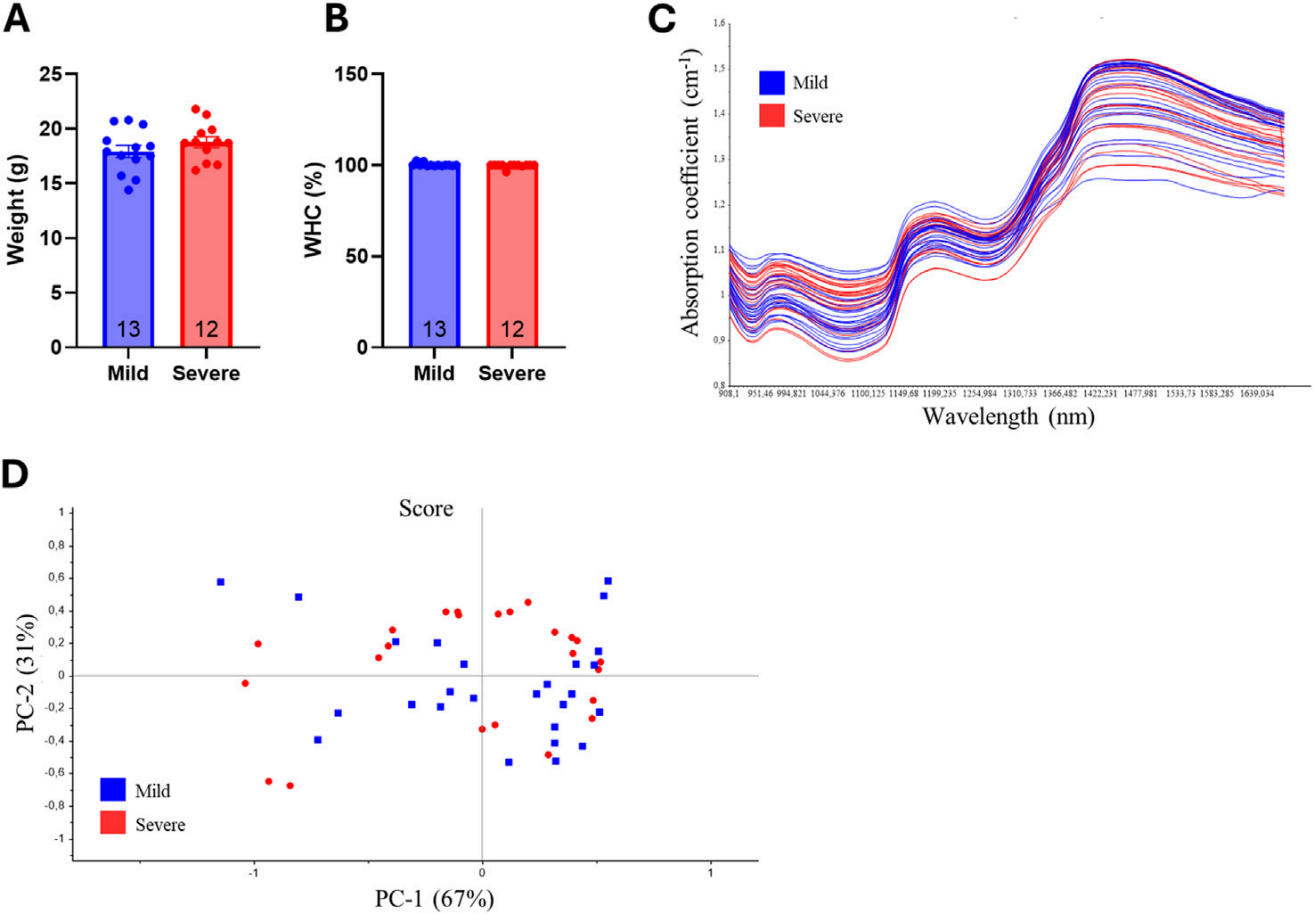
No significant differences in heart weight, water binding, protein, or fat deposition were observed between the hearts of mildly and severely WB-affected chickens. Ross 308 chicken heart **(A)** weight and **(B)** water binding capacity between mild and severely affected wooden-breast hearts (n = 12–13). Data are presented as mean ± SEM. Comparison between mild and affected groups was assessed with Welch`s testing **(C)** Near-infrared spectroscopy and **(D)** principal component analysis of chicken heart samples. Blue, mildly affected; red, severely affected (n = 12).

We have previously shown that severe versus mild WB-affected breasts display lower water-binding capacity (WHC), and differences in fat deposition (Pejšková et al., 2023). To investigate whether the same differences were present in the hearts of chickens affected by WB, hearts from the same broilers were grouped into the same classifications as the breasts (Pejšková et al., 2023). We first assessed differences in the total heart weight. Heart weight was not altered between mild and severe groups, indicating no difference in hypertrophy between the two groups (Figure 1A). Similarly, WHC did not differ between the hearts of mild and severely WB-affected chickens (Figure 1B). Near-infrared spectroscopy (NIR) revealed that there were no differences in water, protein or fat deposition in the hearts between the groups (Figure 1C). Principle component analysis (PCA) confirmed that, compared to the chicken breast (Pejšková et al., 2023), mild and severely affected groups did not display distinct chemical or physical differences (Figure 1D). Altogether, compared to breasts from broilers with severe versus mild WB, hearts from the same animals did not display statistically significant differences in weight, WHC, or fat and protein deposition.

### 3.2 Wooden breast disease severity was not associated with cardiac fibrosis

**FIGURE 2:**
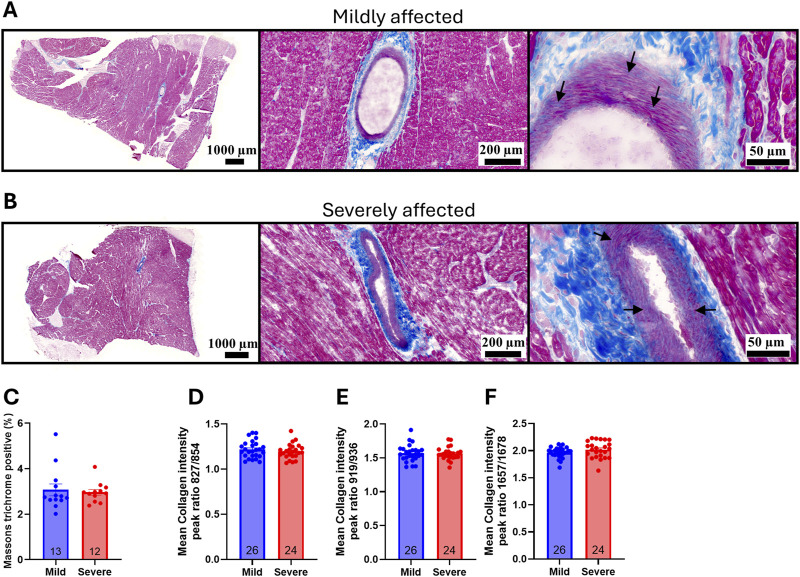
WB disease severity was not associated with cardiac fibrosis. Representative Masson`s trichrome staining of **(A)** mild and **(B)** severely affected Ross 308 chicken left ventricles (n = 12–13). Arrows point to collagen deposition in the tunica media **(C)** Quantification of percentage collagen in the total area of the left ventricle (n = 12–13). Raman spectroscopy collagen markers by peak intensity ratios at **(D)** 827/854 cm^−1^ **(E)** 919/936 cm^−1^, or **(F)** 1,657/1,678 cm^−1^ (n = 12–13, samples run in duplicates). Data are presented as mean ± SEM. Comparison between mild and affected groups was assessed with a Mann-Whitney *U*-test or Welch’s t-test.

One of the hallmarks of WB is the development of fibrosis and changes in the organization of collagen, contributing to the stiffening of the tissue (Sihvo et al., 2014; Sanden et al., 2021). To examine whether WB was also associated with cardiac fibrosis, left ventricle (LV) sections were stained with Masson’s trichrome to identify myocardial tissue (pink) and collagen (blue). Visually, prominent collagen deposition was only observed around and within the larger coronary vessels, with circumferentially aligned fibers present in the tunica media (Figures 2A, B; arrows). The percentage of collagen (blue) in the total area was subsequently quantified, revealing no significant differences in fibrosis in LV tissue between the groups (Figure 2C). Further analysis of collagen accumulation in the hearts with Raman spectroscopy revealed no significant differences in collagen intensity ratios at 827/854 cm^−1^ (Figure 2D), 919/936 cm^−1^ (Figure 2E), or 1,657/1,678 cm^−1^ (Figure 2F), suggesting the levels and distribution of collagen were similar between the groups. Overall, while we and others have previously identified marked changes in fibrosis and collagen organization in WB-affected breasts (Sanden et al., 2021; Pejšková et al., 2023), the same alterations were not present in the heart.

### 3.3 Few alterations in extracellular matrix constituents were observed in the hearts of wooden breast-affected chickens

**FIGURE 3:**
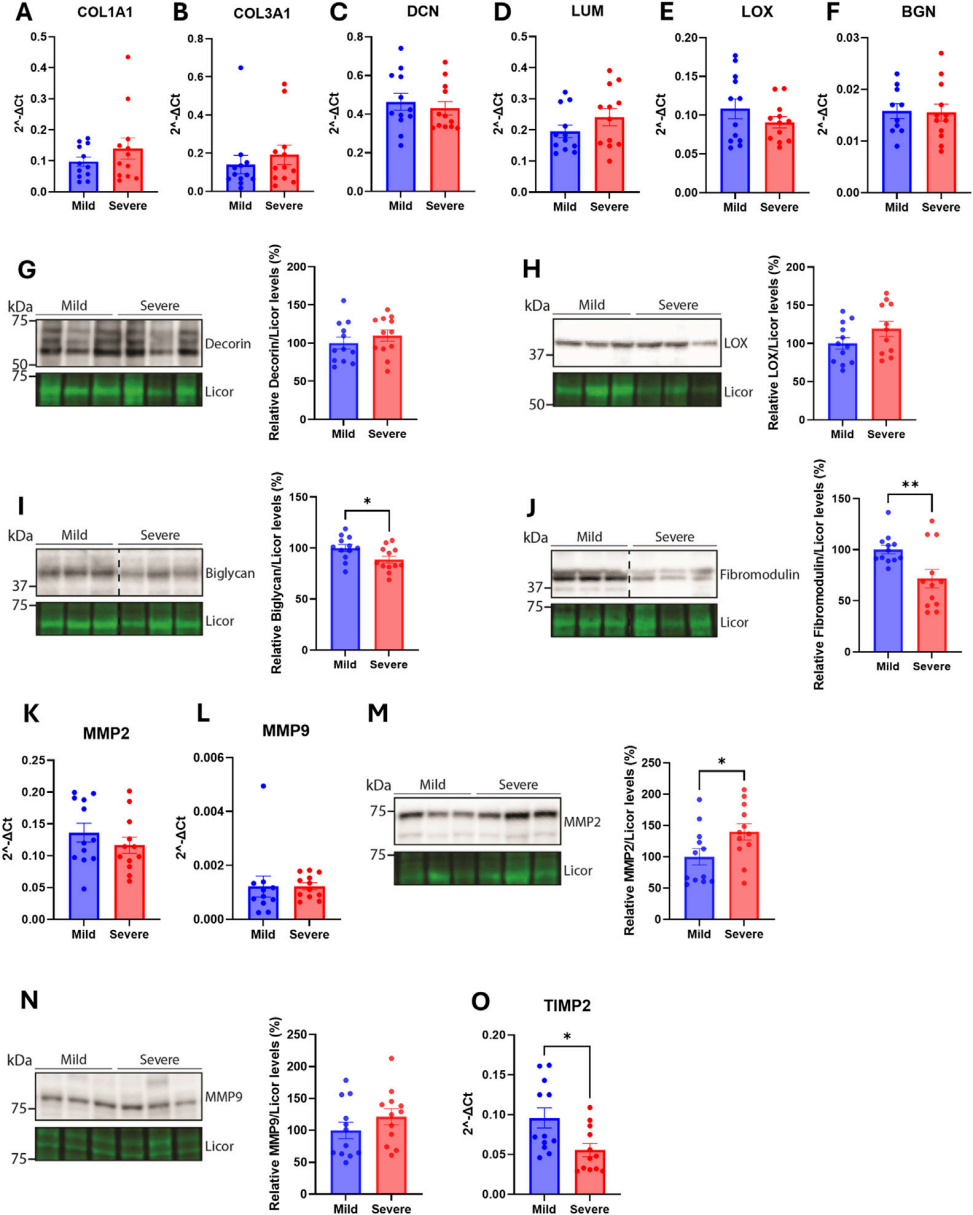
Few alterations in extracellular matrix constituents were observed in the hearts WB-affected chickens. Gene expression by RT-qPCR of **(A)** collagen type 1 α1 **(B)** collagen type 3 α1 **(C)** decorin **(D)** lumican **(E)** LOX, and **(F)** biglycan in hearts of mild and severely affected chicken (n = 12). Immunoblotting of **(G)** decorin **(H)** LOX **(I)** biglycan, and **(J)** fibromodulin in hearts of mild and severely affected chickens (n = 11–12). Gene expression by RT-qPCR of **(K)** MMP2, and **(L)** MMP9. Immunoblotting of **(M)** MMP2 and **(N)** MMP9 in hearts of mild and severely affected chickens (n = 12) **(O)** Gene expression by RT-qPCR of TIMP2 in hearts of mild and severely affected chickens (n = 12). RT-qPCR data are presented as the fold change average relative to the mean of the mildly affected samples and immunoblots are presented as mean ± SEM. Licor was used to show equal loading in **(G–J, M, N)** (lower panels). Differences between groups were assessed with either Welch’s t-tests, Mann-Whitney *U* tests, or student’s *t*-tests.

The lack of fibrosis in the chicken hearts suggests that the overall extracellular matrix (ECM) in the left ventricle is unaffected by WB, independent of the severity. To further analyze the composition of the ECM, several extracellular matrix proteins, cross-linking proteins, and MMPs were analyzed. The mRNA levels of collagen 1A1, collagen 3A1, decorin, lumican, LOX and biglycan were not altered between hearts from mild and severe WB-affected chickens (Figures 3A–F). Consistently, the protein levels of decorin, and LOX were also not altered between groups (Figures 3G, H). The levels of biglycan and fibromodulin were, however, reduced in the hearts of severe versus mildly WB-affected chickens (Figures 3I, J). Although the mRNA levels of the MMPs, MMP2 and MMP9, were not altered (Figures 3K, L), the protein levels of MMP2, but not MMP9, was increased in the hearts of severe WB-affected chickens (Figures 3M, N). The mRNA level of tissue inhibitor of metalloproteinase 2 (TIMP2) was decreased (Figure 3O).

Altogether, although few alterations were detected in the hearts between the two groups, levels of bigylcan, fibromodulin, MMP2 and TIMP2 appeared to be altered.

### 3.4 Syndecan gene expression and protein levels were not altered between hearts from mildly and severely wooden breast-affected chickens

**FIGURE 4:**
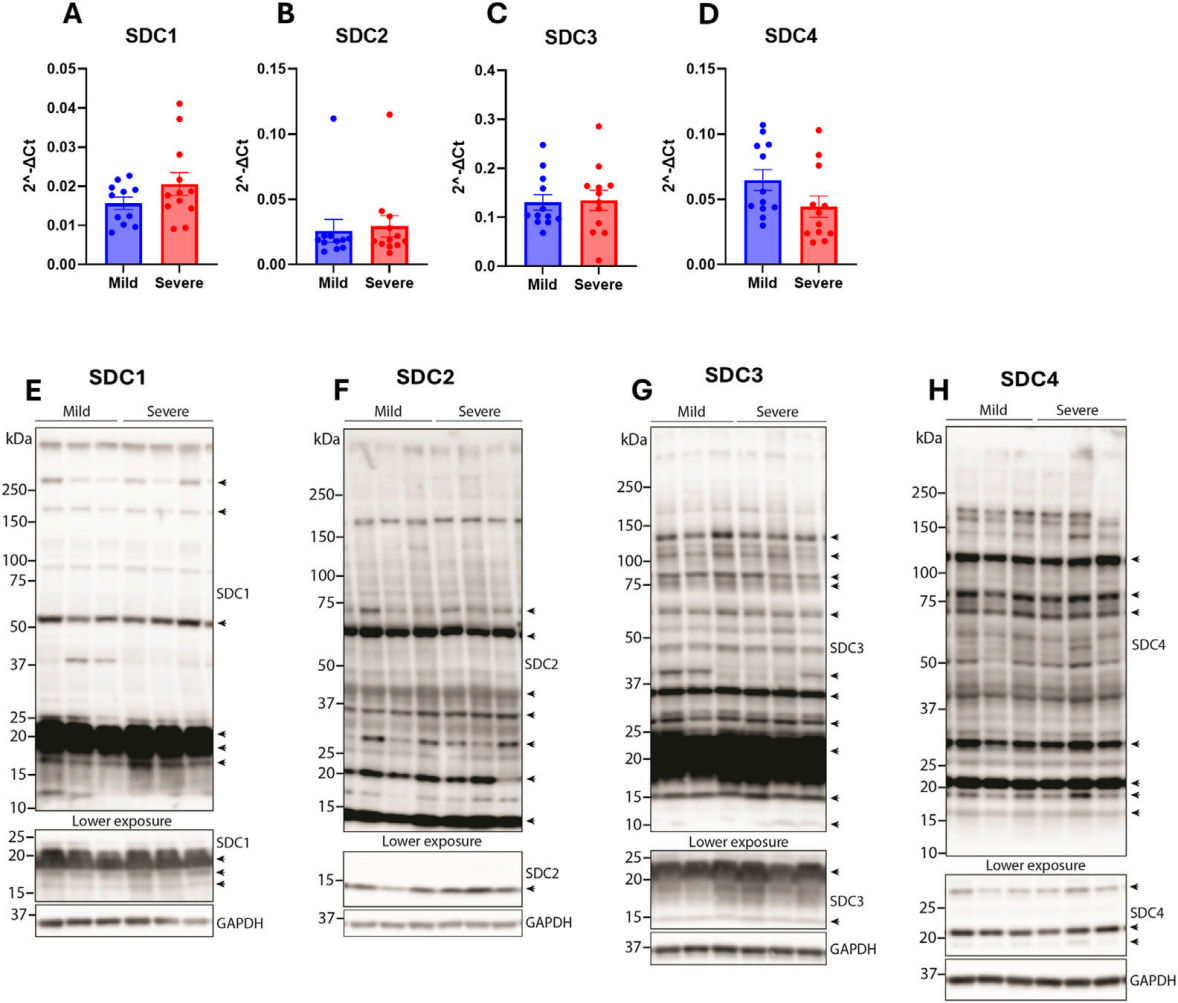
Syndecan gene and protein expression were not altered between hearts from mildly and severely WB-affected chickens. Gene expression of **(A)** syndecan-1 **(B)** syndecan-2 **(C)** syndecan-3, and **(D)** syndecan-4 in the hearts from mild and severely affected chickens was assessed by RT-qPCR (n = 12). Data are presented as the fold change average relative to the mean of the mildly affected samples. Differences between groups were assessed by Welch`s t-tests. Immunoblotting of **(E)** syndecan-1 **(F)** syndecan-2 **(G)** syndecan-3, and **(H)** syndecan-4 in the hearts from mild and severely affected chickens (n = 12). Syndecan-specific bands are annotated with arrows on the right (n = 3). Specificity of bands is shown in Supplementary Figures 1A–D. GAPDH was used to verify equal protein loading (40 µg).

Syndecans have previously been found to be altered in cardiac fibrosis [reviewed in (Lunde et al., 2016)]. Since we have previously found the syndecans to be differentially regulated between the mild and severely WB-affected groups (Pejšková et al., 2023), we investigated the gene expression and protein levels of the syndecans in the hearts of the same broilers. Compared to the chicken breast where syndecan-2 mRNA expression was decreased and syndecan-4 expression was increased in severely affected WB breasts (Pejšková et al., 2023), no differences were observed in the gene expression levels of syndecan-1-4 in the hearts between groups (Figures 4A–D). To investigate potential alterations in the protein and shedding levels of the syndecans, the heart lysates were immunoblotted for the syndecans using custom-made antibodies against the chicken cytoplasmic domains (Pejšková et al., 2023). As expected, we detected multiple syndecan-positive bands (Figures 4E–H). The specificity of these bands was verified by epitope blocking experiments (Supplementary Figures 1A–D), revealing specificity of bands at ∼10–250+ kDa for syndecan-1, ∼10–75 kDa for syndecan-2, ∼10–150 kDa for syndecan-3 and ∼15–150 kDa for syndecan-4 (Figures 4E–H). The core proteins of the chicken syndecans are relatively small where syndecan-1 is 308 amino acids (aa) in length, syndecan-2 is 201 aa, syndecan-3 is 405 aa, and syndecan-4 is 197 aa. The smaller molecular weight fragments (<20 kDa) observed in syndecan-1-4 are likely the cytoplasmic tail and transmembrane domain of the syndecans after extracellular shedding. We also observed multiple higher molecular weight bands which are likely SDS-resistant homo- or hetero-oligomers of the syndecans. We did, however, not observe significant differences in the levels of the different syndecan forms between hearts from mild and severely WB-affected chickens (Figures 4E–H).

### 3.5 Alterations in Wnt and mTOR signaling, HSP70, and MLP

**FIGURE 5:**
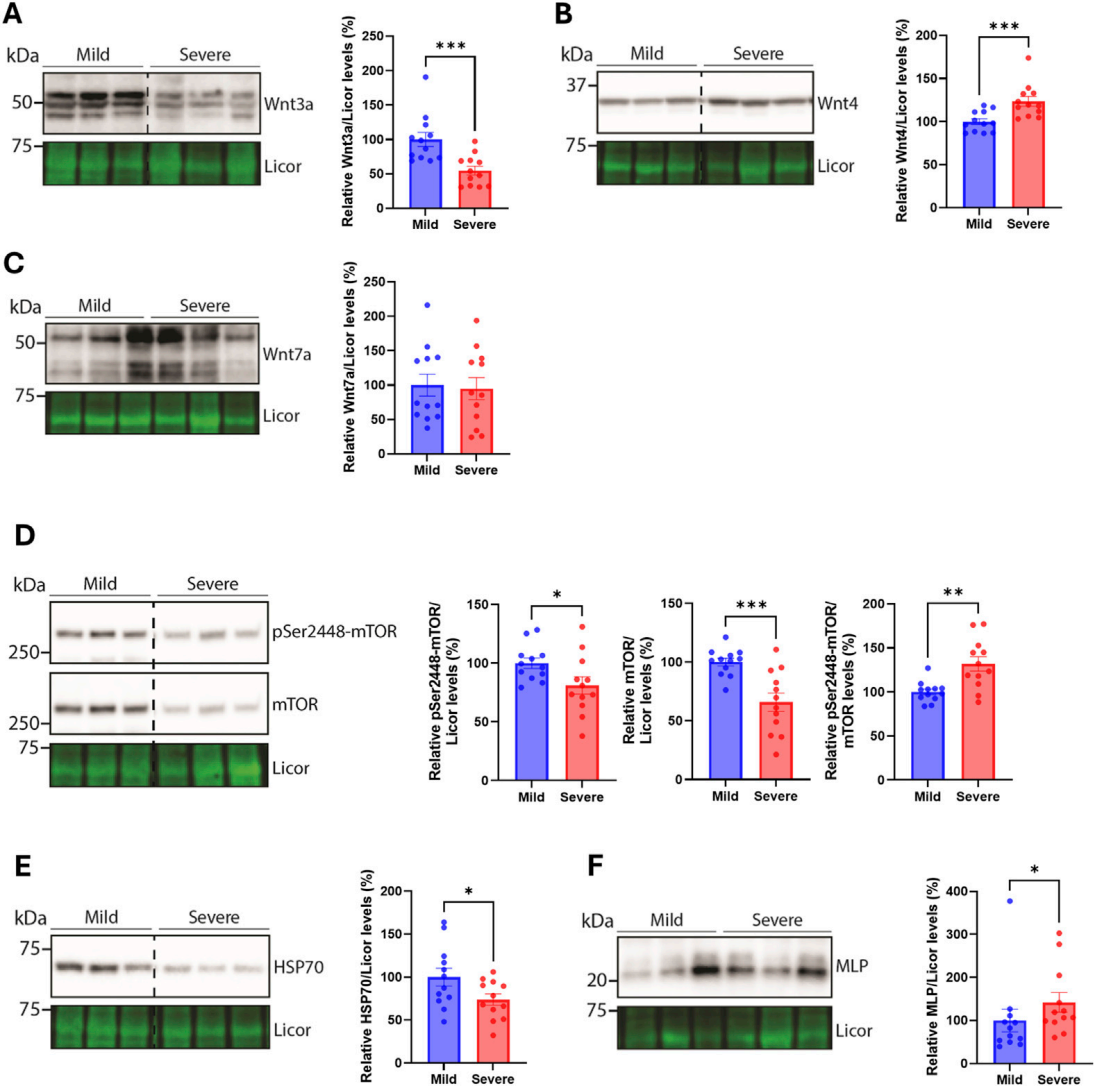
Alterations in Wnt and mTOR signaling, HSP70, and MLP. Immunoblotting of **(A)** Wnt3a **(B)** Wnt4 **(C)** Wnt7a **(D)** pSer2448-mTOR and mTOR **(E)** HSP70, and **(F)** MLP in hearts from mild and severely affected chicken hearts (n = 12). Licor was used to show equal loading (lower panels). Immunoblots are presented as mean ± SEM. Differences between groups were assessed with Mann-Whitney *U*-tests or student’s *t*-tests.

To assess similarities between the breast and the heart of severely versus mildly WB-affected chickens, we next analyzed various signaling pathways that we have previously found altered (Pejšková et al., 2023). The protein level of the Wnt signaling ligand Wnt3a was reduced in the hearts of the severely WB-affected chickens (Figure 5A), while the levels of Wnt4 were increased (Figure 5B). Levels of Wnt7a remained unaltered between the groups (Figure 5C). We have previously found increased levels of both Wnt4 and Wnt7a in the severely WB-affected chickens (Pejšková et al., 2023). Furthermore, the mammalian target of rapamycin (mTOR) signaling also appeared to be altered in the hearts of severe WB-affected chickens. Whereas both the pSer2448-mTOR and total mTOR levels were reduced, the pSer2448-mTOR/mTOR level was increased (Figure 5D).

The heat shock protein HSP70, known to be increased in the blood of chickens upon heat stimulation (Greene et al., 2024), was also reduced in the hearts of severely WB-affected chickens (Figure 5E). HSP70 is also located in the mitochondria where it acts as an important chaperone of the import, folding, and assembly of proteins in the mitochondria (Herrmann et al., 1994). The small muscle LIM protein (MLP), known for its crucial role in cardiac and skeletal function [reviewed in (Vafiadaki et al., 2015)] was elevated in the hearts of severe WB-affected chickens (Figure 5F). Other signaling pathways, such as Akt and p38 mitogen-activated kinase (MAPK) phosphorylation and total protein levels, and markers of cardiomyocyte contractile health such as cardiac troponin T, were not altered between the WB-affected groups (Supplementary Figures 2A–C). We also analyzed the expression of known markers of inflammation, the cytoskeleton, myofibroblast, and fibrosis-triggering genes. No differences were observed in interleukin-1β (IL-1β), toll-like receptor 4 (TLR4), tubulin α1 (TUBA1A), tubulin β1 (TUBB1), desmin (DES), β-tropomyosin (TPM2), cardiac troponin T (TNNT2), platelet-derived growth factor receptor β (PDGFRb), α-actin (ACTA2), or transforming growth factor β1 (TGFB1) gene expression between hearts of the mildly and severely WB-affected chickens (Supplementary Figures 3A–J).

### 3.6 Functional enrichment of RNA sequencing

Finally, since few alterations were found in the macronutrient composition of the hearts, fibrosis, ECM gene expression and protein levels, and classic disease-associated cardiac markers, we performed RNA sequencing to identify potential alterations between hearts from mildly and severely WB-affected chickens. The number of differentially regulated genes between the two groups was minimal, further confirming the absence of a strong pathological signature in the hearts of WB-affected chickens.

**FIGURE 6:**
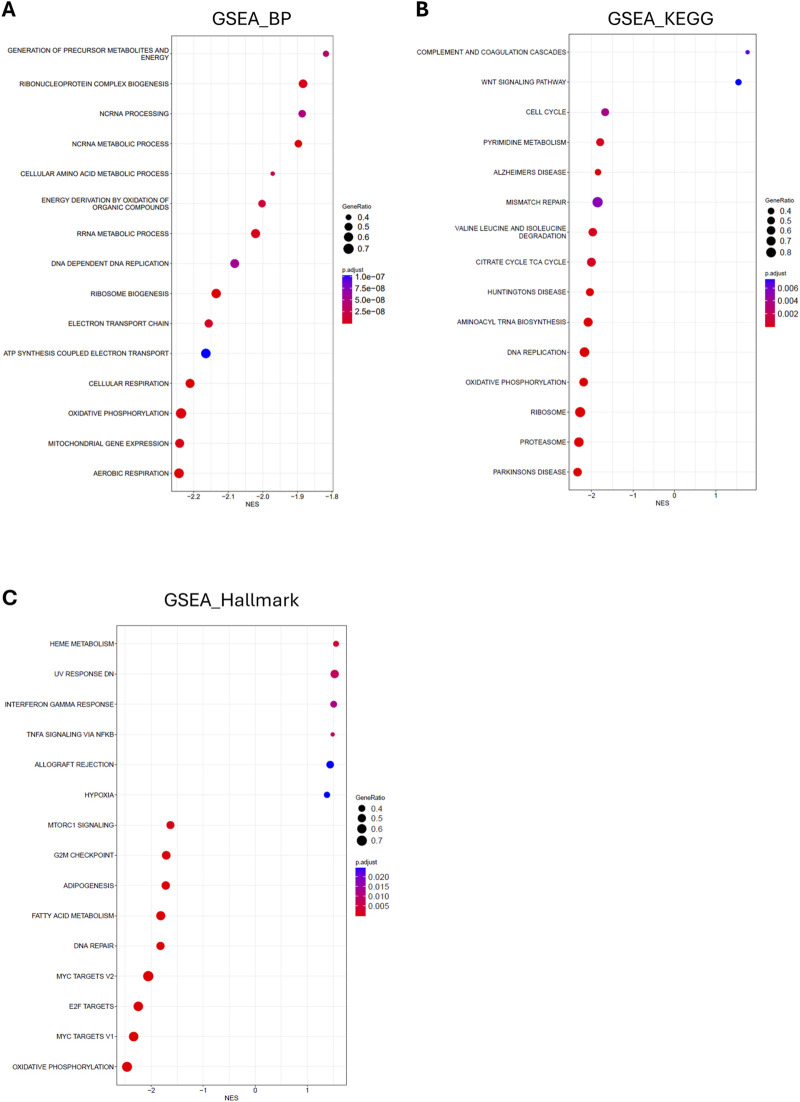
Functional enrichment of RNA sequencing of hearts from mild and severely WB-affected chicken. Bubble plot of gene set enrichment analysis of the top 15 altered **(A)** gene ontology biological processes **(B)** KEGG pathways, and **(C)** Hallmark pathways between hearts from mild and severely WB-affected chickens. Dot size indicates the number of genes in each given function. Dot color indicates the p-adjust for each given function as reported in the indicated scale. The X-axis shows the normalized enrichment score (NES) of the differentially expressed processes and pathways in hearts from severe versus mild WB-affected chickens.

However, functional gene set enrichment analysis (GSEA) of the global changes in gene expression between mildly and severely affected chickens, identified an interesting trend: Functions associated with inflammation and acute phase response were enriched with a positive Normalized Enrichment Score (NES) in severely WB-affected chickens, while functions associated with cell proliferation and metabolism were enriched with a negative NES (Figures 6A–C).

## 4 Discussion

In the present study, we have investigated the histological and molecular alterations in the hearts of mildly and severely WB-affected chickens. While we have previously found alterations in ECM remodeling, fibrosis development, syndecan expression and shedding, and various signaling pathways in severely affected chicken breasts (Pejšková et al., 2023), the hearts from the same chickens did not display changes to the same extent. Although we observed a reduction in biglycan and fibromodulin, an increase in MMP2, and alterations in Wnt ligands, mTOR, HSP70, and MLP, few differences in total heart weight, nutritional composition, fibrosis, ECM, or cardiac and inflammatory markers were detected. However, functional enrichment analysis of RNA sequencing of the hearts suggests a different molecular footprint between the groups.

WHC, NIR, and PCA analyses were conducted to analyze macronutrient composition for a larger overview of the hearts between severity groups. Previous studies of WB-affected breasts have shown a decrease in protein content and water binding, but an increase in fat and collagen deposition in severely affected samples (Soglia et al., 2016; Wold et al., 2017; Pejšková et al., 2023). However, the hearts from severely WB-affected chickens did not display alterations in their macronutrient composition compared to samples from mildly affected chickens. Fat accumulation in the heart is known to initiate an inflammatory response, the production of reactive oxygen species, and a decline in mitochondrial function, which can harm the heart’s function (Guzzardi and Iozzo, 2011). Consistently with no fat or collagen deposition, we did not detect changes in the gene expression levels of markers associated with inflammation, such as IL-1β, TLR4, or TGFβ.

Hypertrophy of the heart is often associated with fibrotic development and remodeling of the ECM of the myocardium in mammalian disease (Li et al., 2018). Progressive fibrotic remodeling is linked to a decline in cardiac function, an increased risk of arrhythmias, and eventually heart failure (de Jong et al., 2011; Travers et al., 2016; Park et al., 2019). Based on the histological appearance, fibrosis can be categorized into three subtypes: replacement, vascular, or interstitial fibrosis. In the chicken breast, fibrosis as a result of WB disease seems to be mainly interstitial fibrosis, where an increase in ECM proteins expands the space between the myocytes (Che et al., 2022). However, the infiltrative interstitial fibrosis observed in the breast muscle of WB chickens (Soglia et al., 2016) was not evident in the myocardial tissue of mildly and severely affected chickens in this study. We did, however, observe pronounced fibrillar collagen fiber formation within the tunica media of the coronary arteries in both groups. These fibers were circumferentially aligned, a pattern more commonly observed in arteries during physiological aging (Jadidi et al., 2021) and associated with increased arterial wall stiffness. Given the lack of fibrotic development and other indicators of pathological cardiac remodeling, the pronounced but organized collagen fiber deposition within the coronary vessel may indicate an adaptive development of stabilizing fibers within the heart. Indeed, the radically accelerated growth rate of commercial broilers compared to earlier unselected strains has resulted in larger body mass with a relative smaller heart mass (Harash et al., 2019). It could be postulated that this adds additional strain on the vasculature of these chickens, including the aorta itself, which may require additional structural support in the form of stabilizing fibrillar collagens in the tunica media, which we observed in our study.

Furthermore, we analyzed Wnt and mTOR signaling, due to our previous findings of dysregulated Wnt and Akt/mTOR in the severely affected breasts of the same broilers (Pejšková et al., 2023). In the heart, Wnt ligands Wnt3a and Wnt4 were decreased and increased, respectively, suggesting the heart of severely versus mildly WB-affected chickens also have altered Wnt signaling. Wnt signaling is important for many embryonic processes such as cell proliferation, spatial tissue patterning and differentiation (Foulquier et al., 2018). In the adult heart, an upregulation in the gene expression of Wnt ligands has been observed after myocardial infarction in mice (Aisagbonhi et al., 2011), and activation of the signaling pathway has been found to promote fibrosis development upon cardiac injury and repair [Reviewed in (Deb, 2014; Pahnke et al., 2016)].

mTOR signaling, on the other hand, is required for hypertrophy development, and its inhibition or deletion leads to a lack of compensatory and pathological hypertrophy, inhibition of protein synthesis, and cardiac dysfunction (Sciarretta et al., 2014). We observed lower levels of pSer2448-mTOR and total mTOR levels, which may indicate that the hearts of severely WB-affected chickens are less able to respond or cope with increased stressors, such as changes in cardiac demand due to an increase in skeletal muscle growth.

The reduction in HSP70 we observed may also suggest that the hearts of severely WB-affected chickens are less robust in coping with prolonged stressors. HSP70 protects the heart from ischemia-reperfusion injury (Song et al., 2020). HSP70 has also been found to trigger hypertrophy and fibrosis (Liu et al., 2019), which we did not observe in the hearts included in our study.

The muscle-specific protein MLP was increased in the hearts of severe WB-affected chickens. MLP has previously been found to be important for chicken satellite cell differentiation, and chicken myofiber composition (Han et al., 2019; Shan et al., 2023). In humans, MLP levels increase in the failing heart (Boateng et al., 2007). Altogether, an increase in MLP levels in the hearts of severe WB-affected chickens may suggest these hearts are more prone to dysfunction later in development.

Lastly, we performed RNA sequencing of hearts from chickens affected by mild and severe WB disease. The finding via GSEA analysis that metabolic functions, especially those related to mitochondrial function, are downregulated, is consistent with our previous findings in skeletal muscle (Pejšková et al., 2023). Also consistent with the early stages of developing heart disease, we observed an increase in inflammatory functions. Altogether, these data suggest that, while at the phenotypic and molecular levels little to no difference is observable between mildly and severely affected chickens, the global transcriptomic landscape appears to be laying the molecular foundation for the development of heart disease.

An important factor to consider in interpreting the presented results is the lack of comparison to a healthy control. However, all chickens of the Ross308 breed seem to display some signs of skeletal muscle myopathy (Pejšková et al., 2023; Pejšková et al., 2024), making the use of a healthy control group difficult. Our study compared cardiac samples from severely affected chickens to those from mildly affected chickens. As such, it is not possible to differentiate whether no changes were present in the heart between the groups, or whether changes were present to the same extent independent of severity. A comparative analysis against another breed, such as the more slow-growing Hubbard JA787 broiler line with a lower mortality rate when bred under commercial conditions, may be necessary to answer such questions (Forseth et al., 2024).

Another alternative is the age at which the broilers were harvested. The chickens used in this study were 36 days post-hatching, which may be too young for cardiac fibrotic remodeling and molecular changes to be present. It is possible that the accelerated growth rate of the skeletal muscle, inducing pathological hypertrophy and fibrotic remodeling, outstrips cardiac growth. This is also evident in Duchenne muscular dystrophy where patients normally develop clinical symptoms of weakness and fatigue in the skeletal muscle of the legs at 3–5 years of age. These patients subsequently develop alterations in their cardiac function at approximately 9–10 years of age (Meyers and Townsend, 2019).

To conclude, we have in this study investigated alterations in the hearts of Ross 308 broiler chickens with mild and severe WB disease. Although few alterations were found in the macro composition of the hearts between the two groups, deeper molecular analysis and RNA sequencing suggest that the molecular footprint does differ depending on WB severity. Such differences may become more prominent with age, perhaps leading to cardiac dysfunction.

## Data Availability

The data presented in the study are deposited in the European Nucleotide Archive (ENA) repository, accession number PRJEB85439.
