# Supplementary figures and images for: Cardiac implications of chicken wooden breast myopathy
Source: Front Physiol. 2025 Mar 5;16:1547661. doi: 10.3389/fphys.2025.1547661 (PMC11919848; doi:10.3389/fphys.2025.1547661)

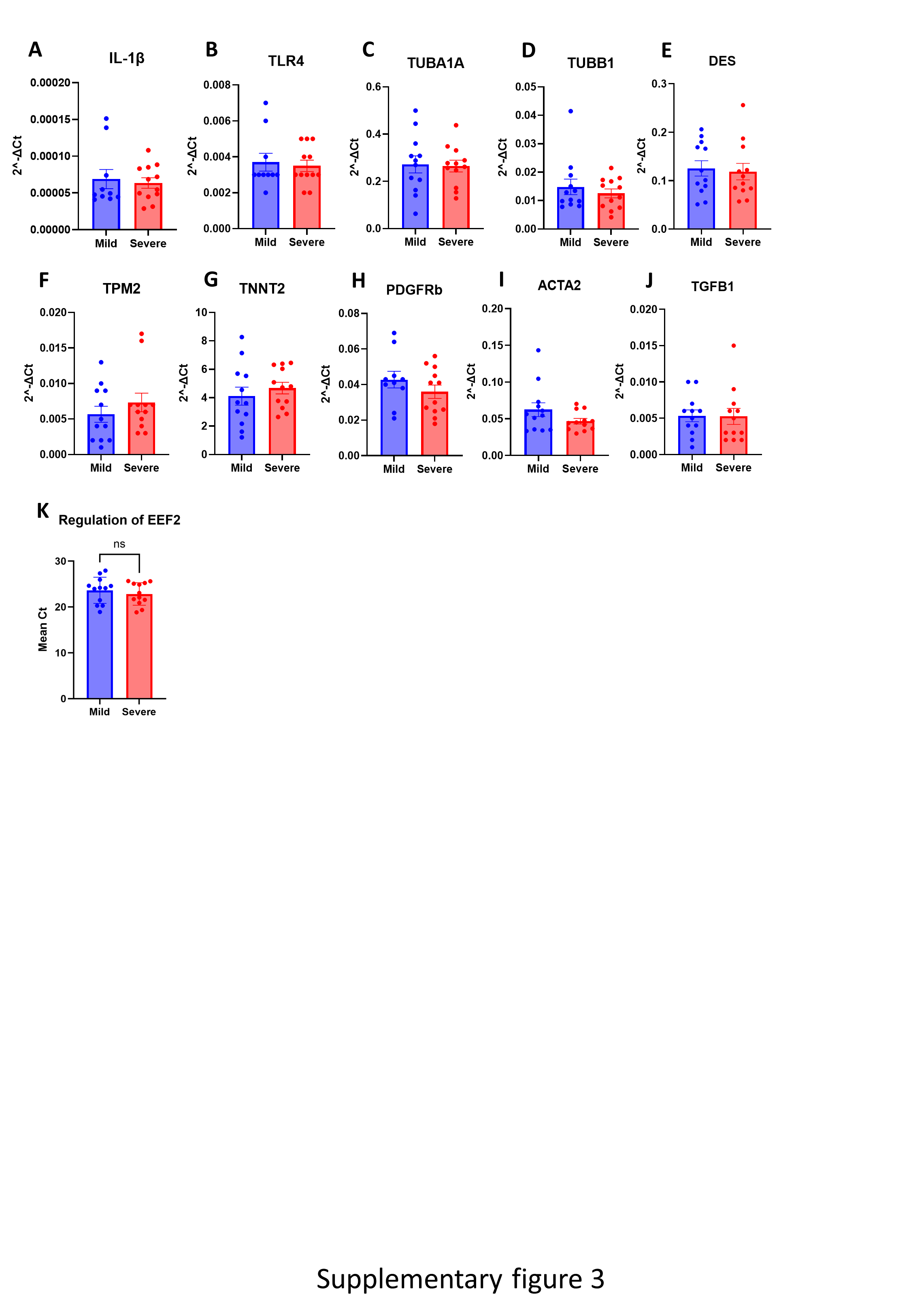

Supplement: Supplementary file 1 [file Image3.tif]

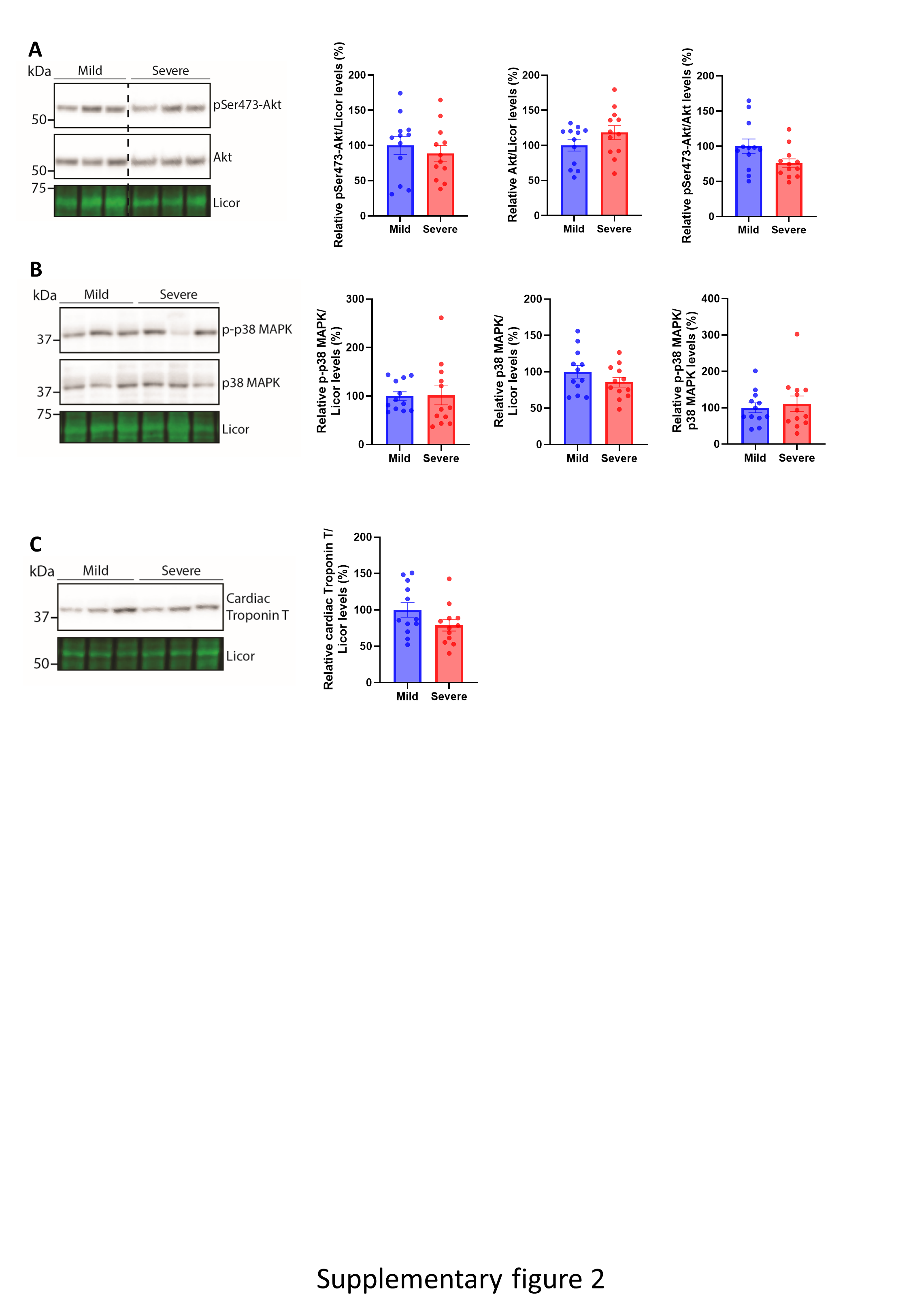

Supplement: Supplementary file 2 [file Image2.tif]
