# Supplementary material for: Cardiac implications of chicken wooden breast myopathy
Source: Front Physiol. 2025 Mar 5;16:1547661. doi: 10.3389/fphys.2025.1547661 (PMC11919848; doi:10.3389/fphys.2025.1547661)

Full length blots for figure 3G

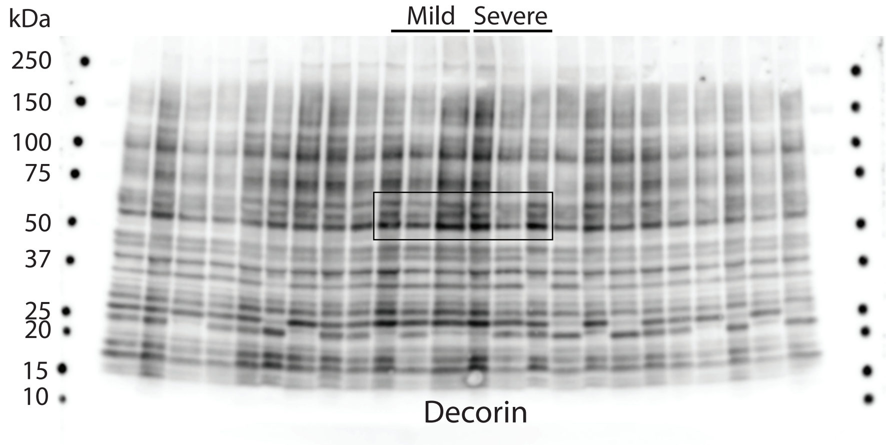

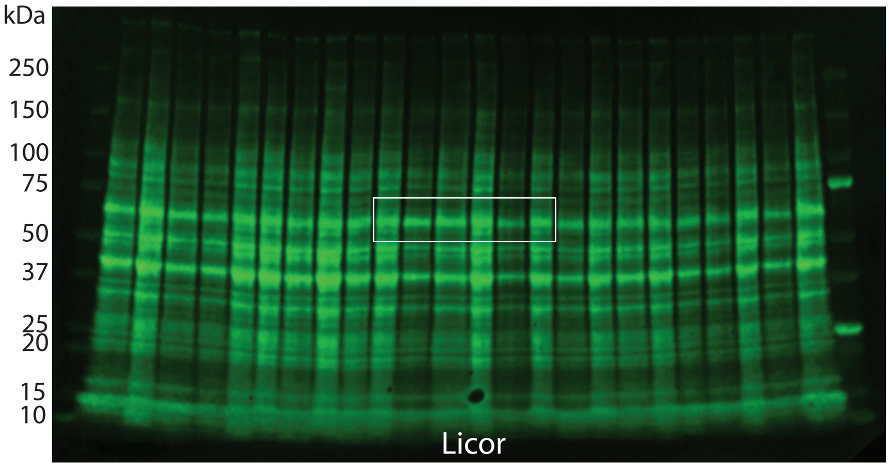

Full length blots for figure 3H

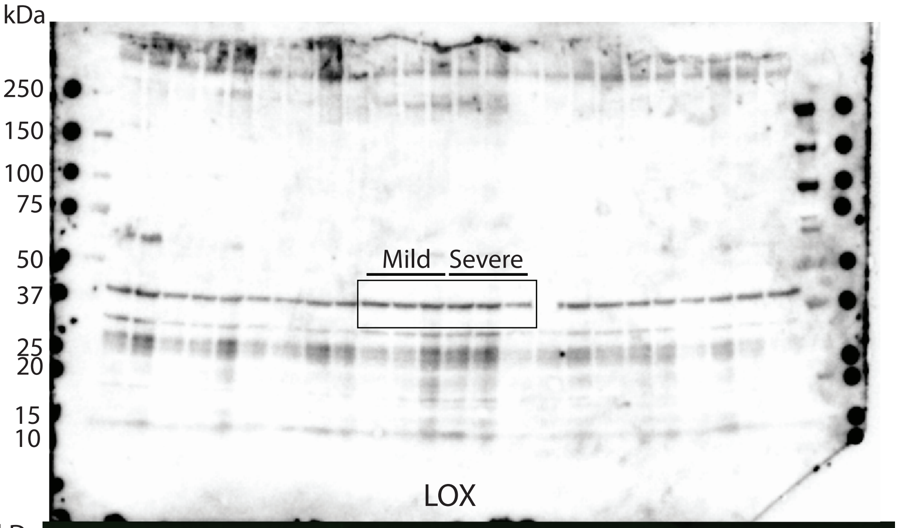

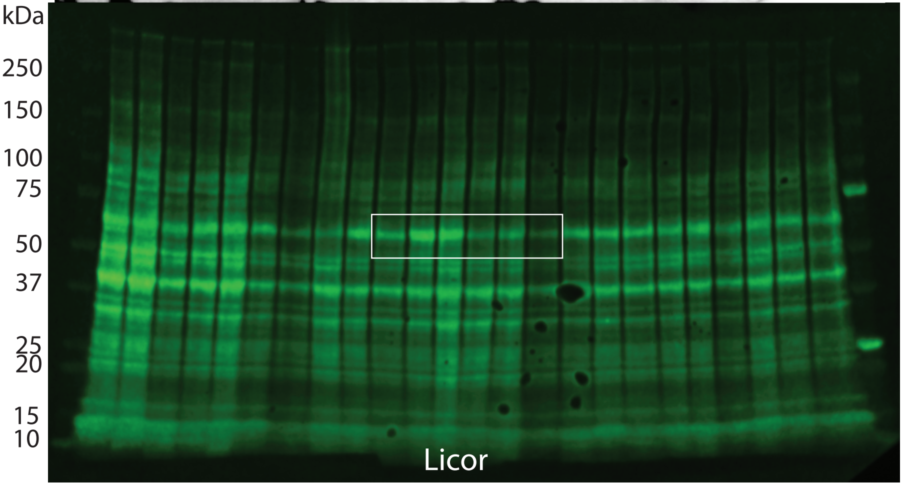

Full length blots for figure 3I

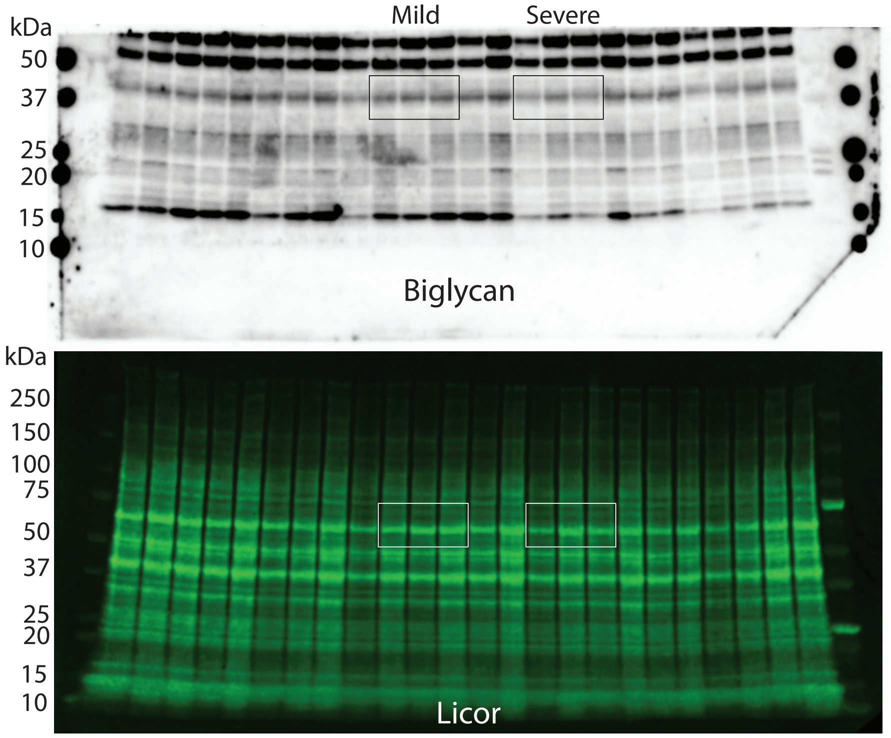

Full length blots for figure 3J

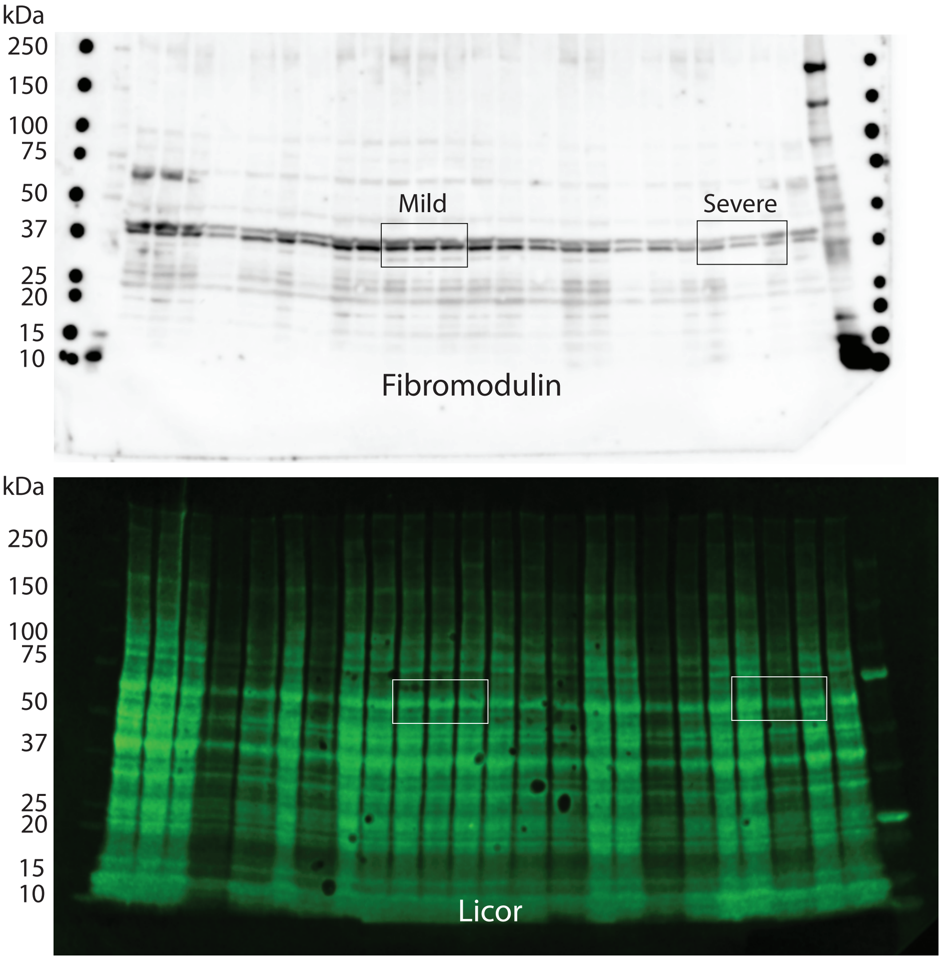

Full length blots for figure 3M

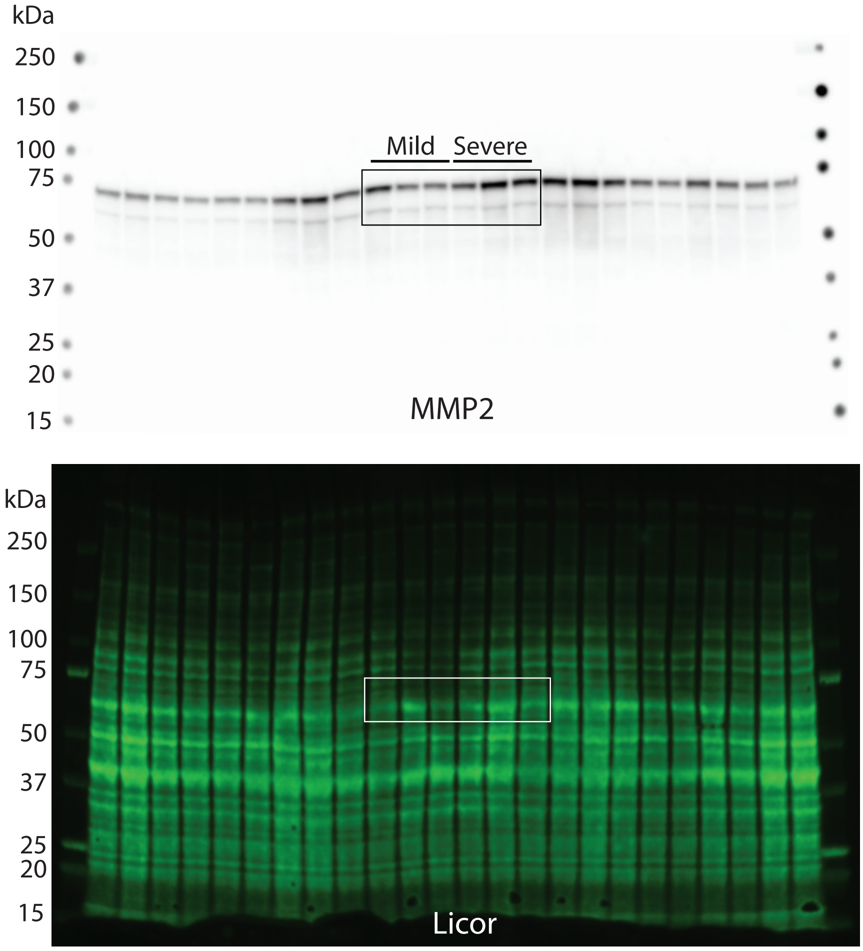

Full length blots for figure 3N

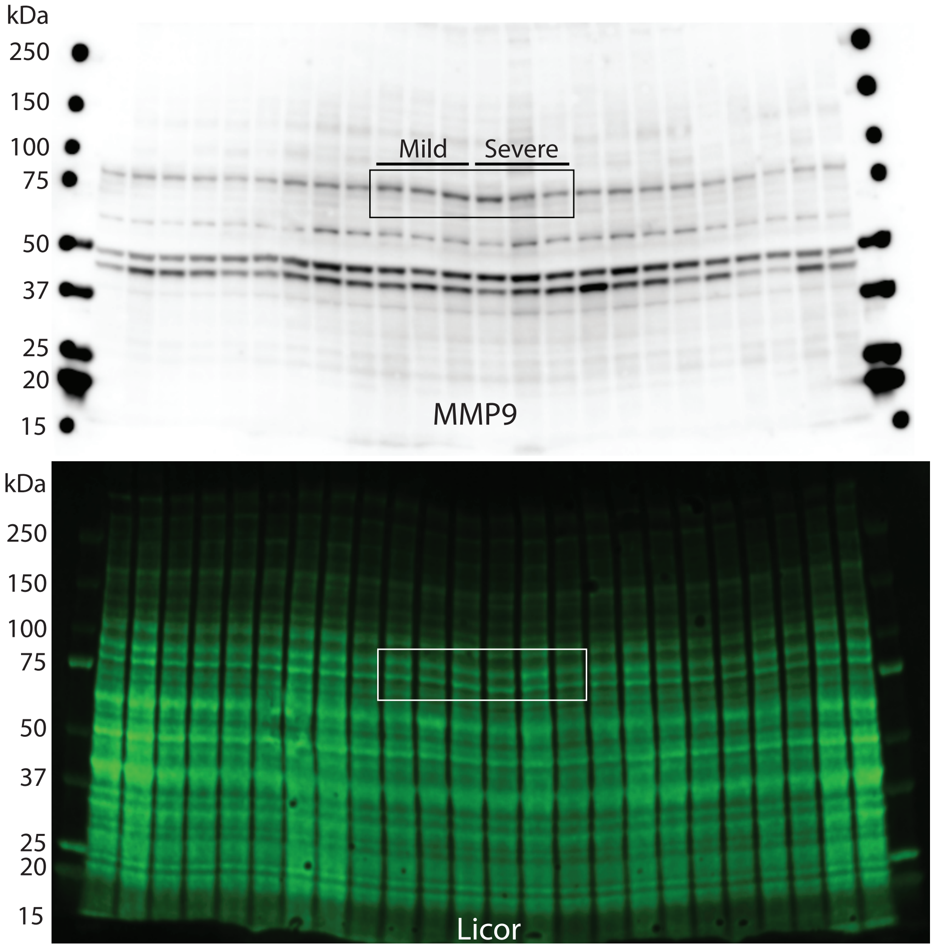

Full length blots for figure 4E

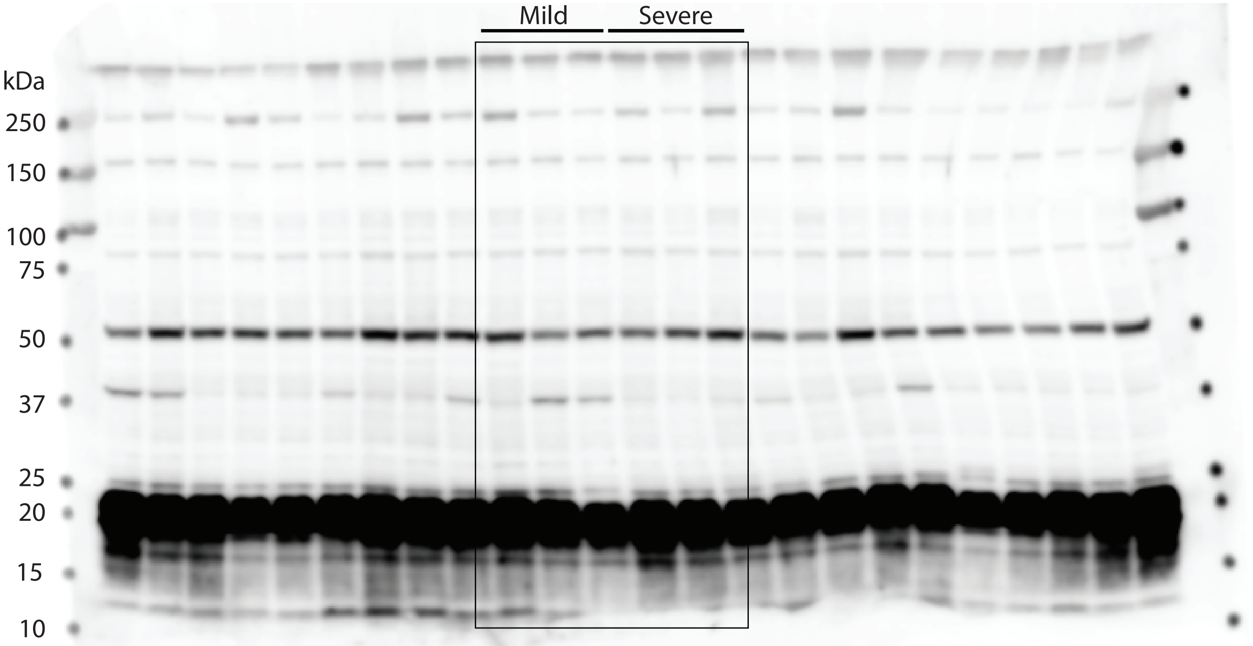

SDC1

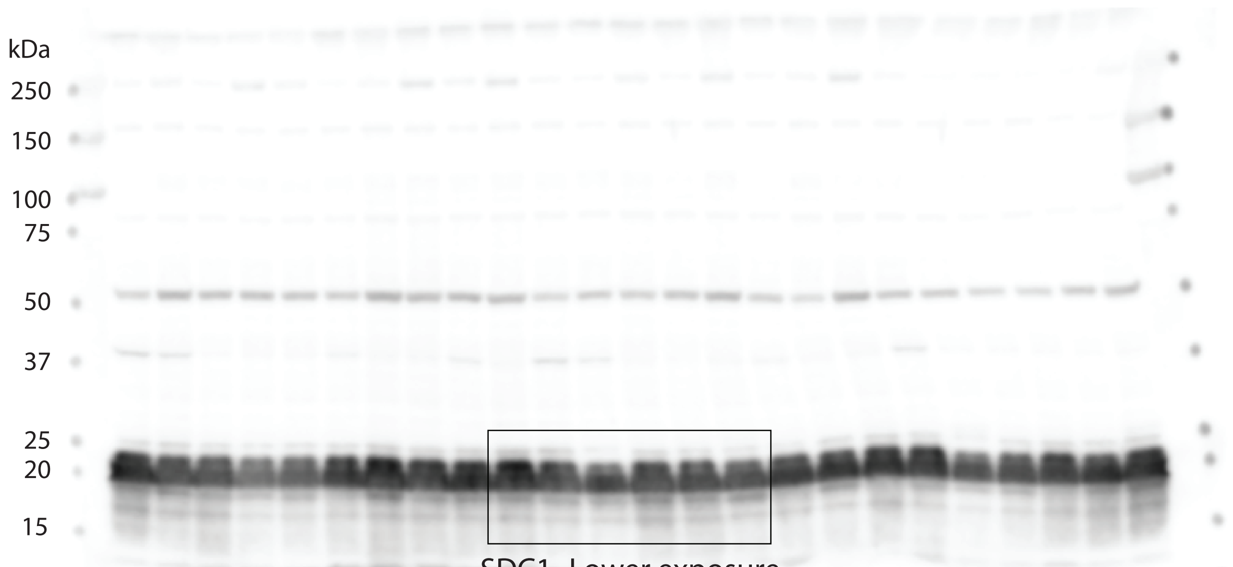

SDC1- Lower exposure

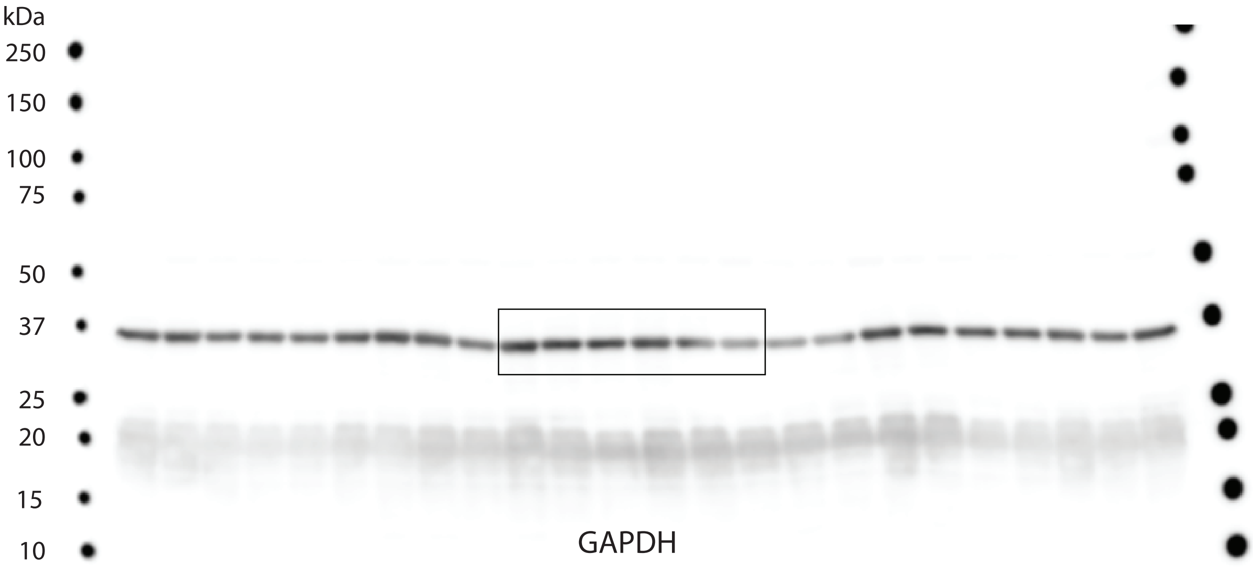

GAPDH

Full length blots for figure 4F

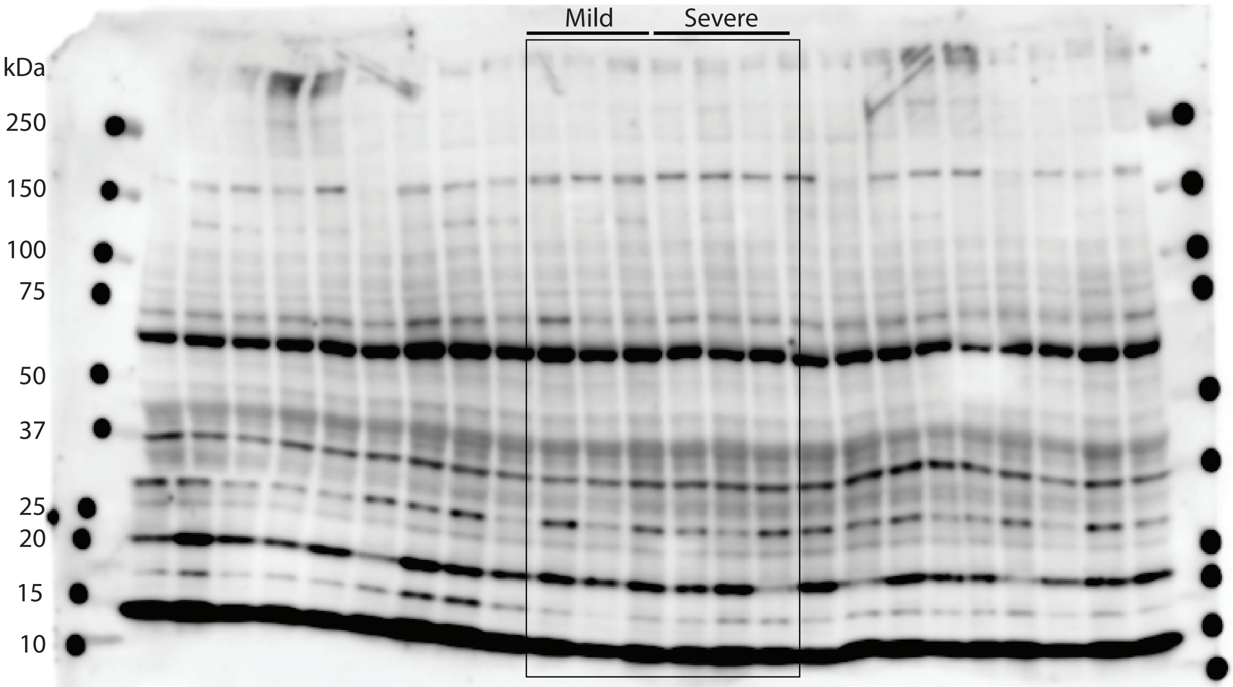

SDC2

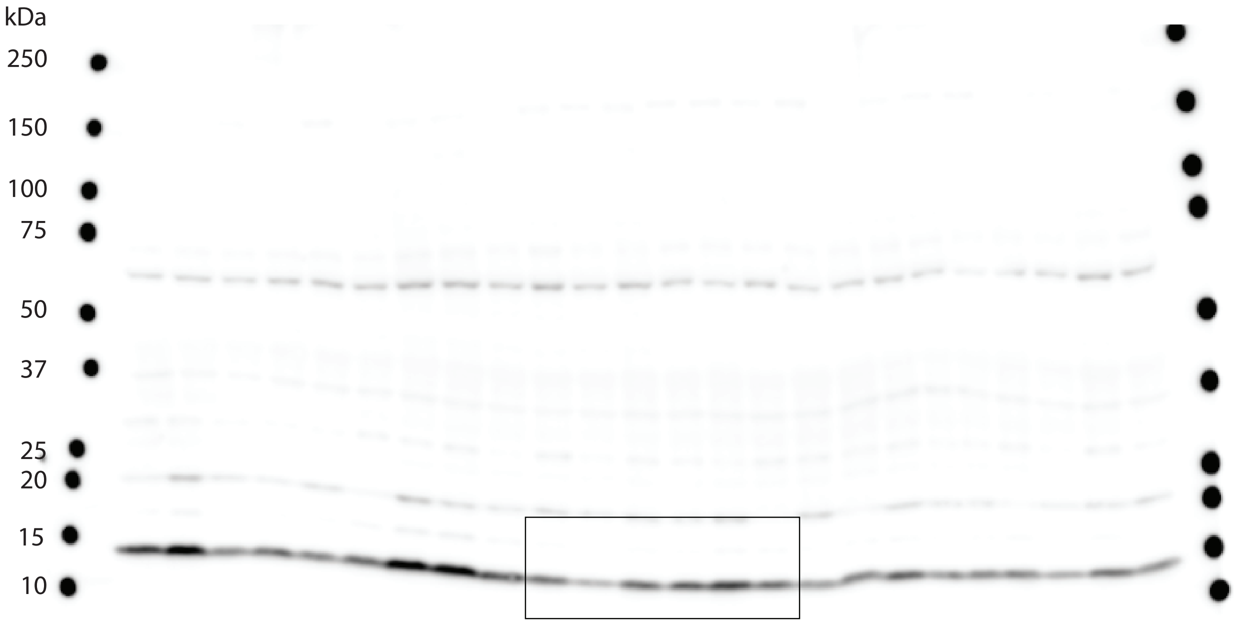

SDC2- Lower exposure

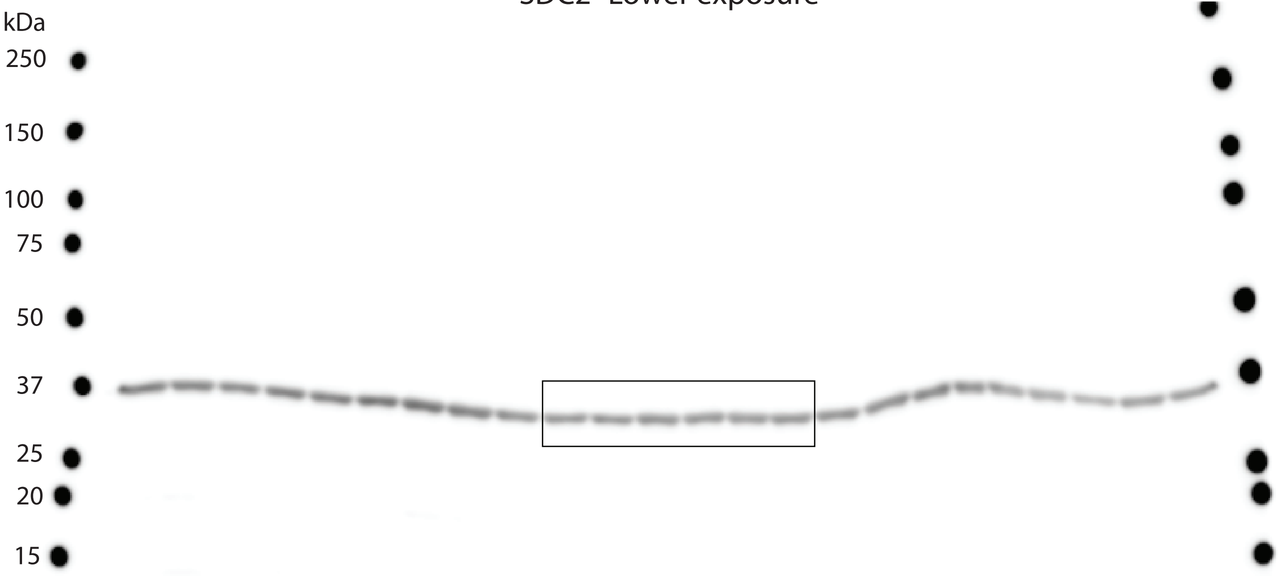

GAPDH

Full length blots for figure 4G

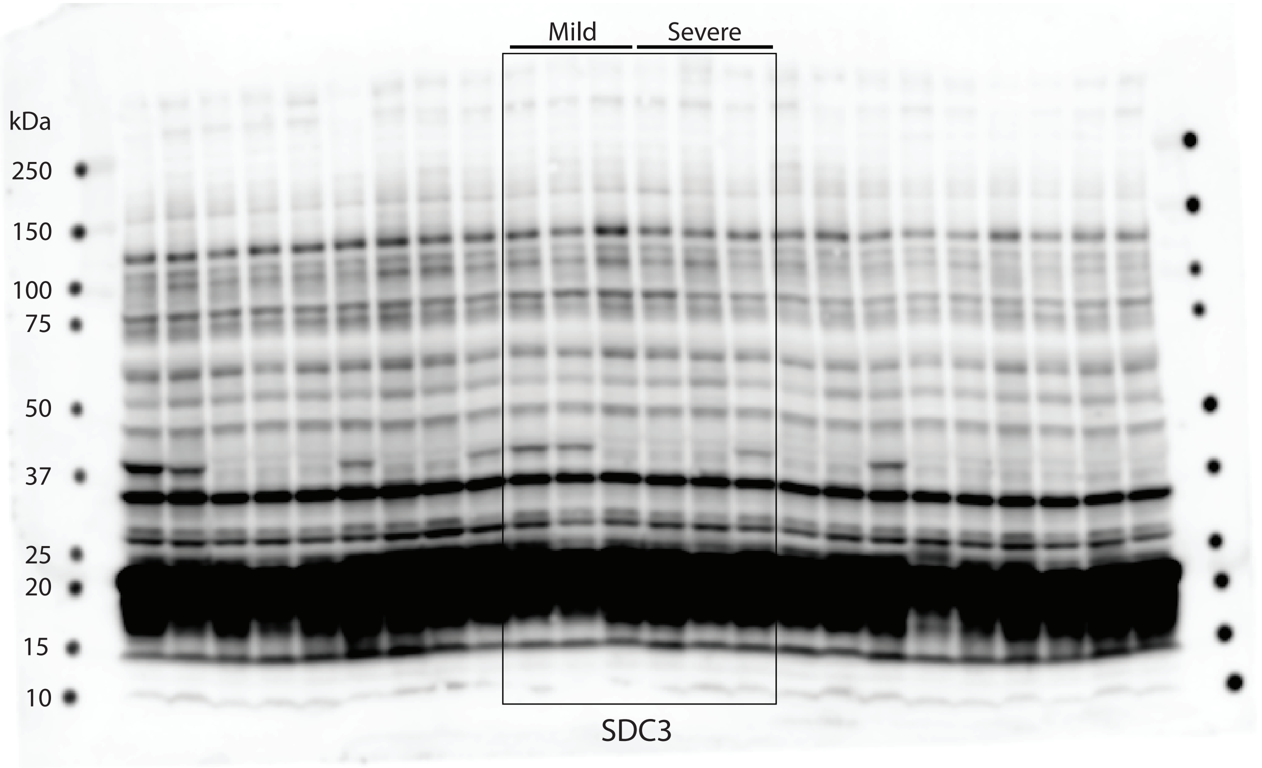

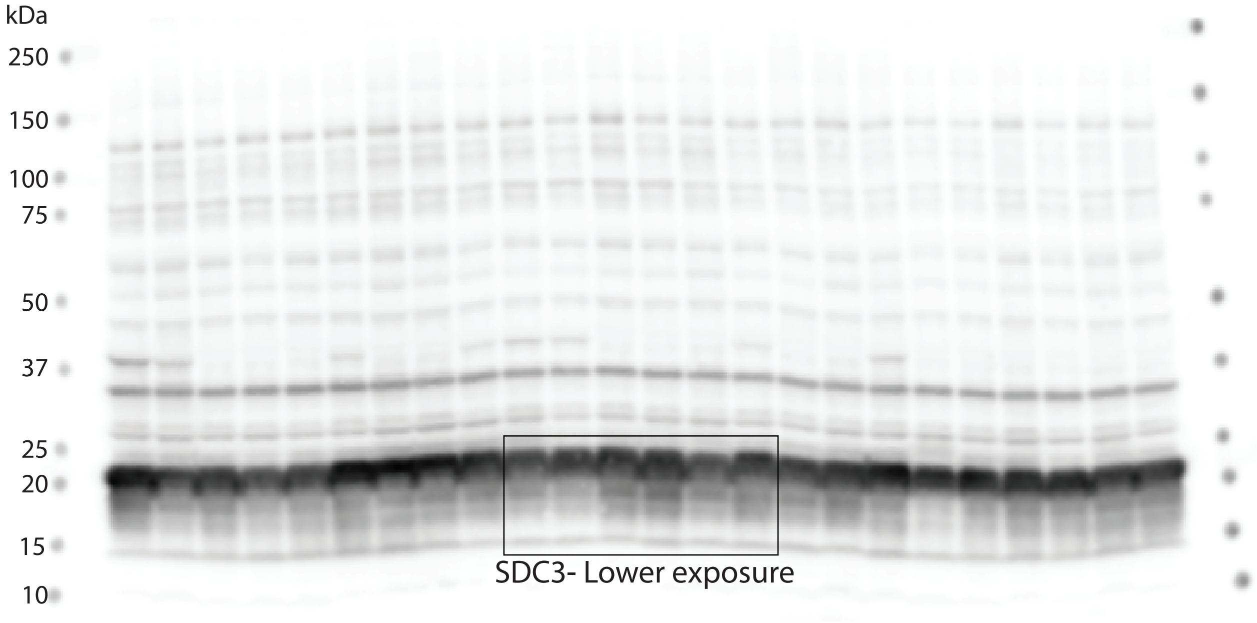

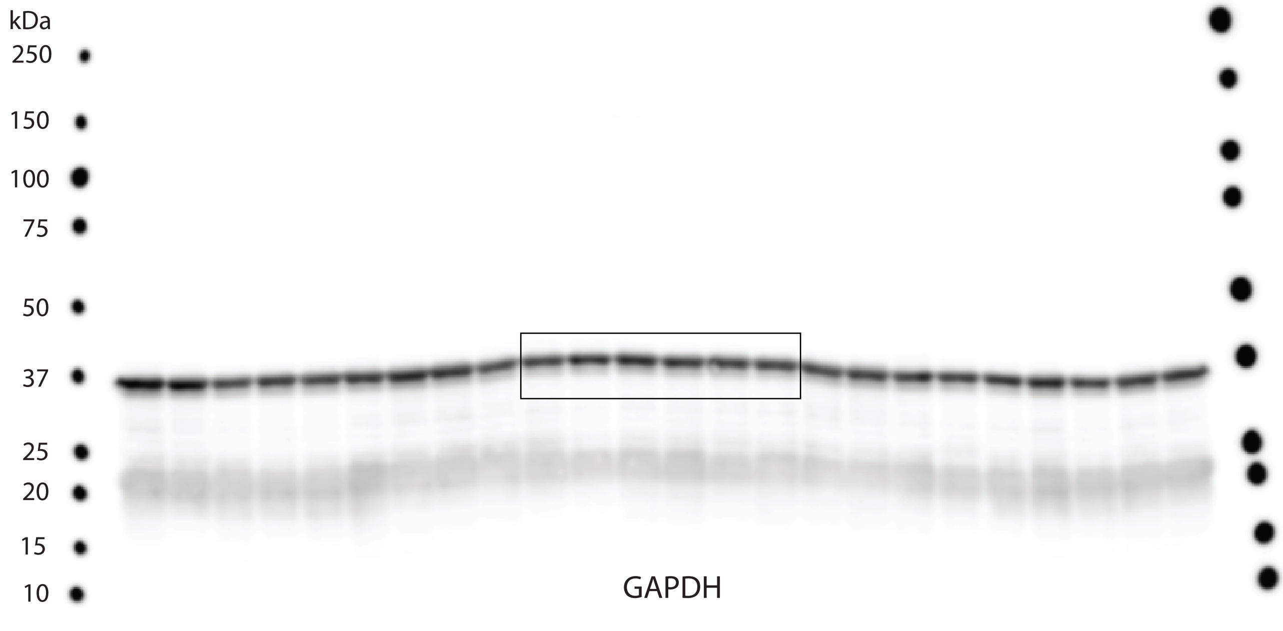

Full length blots for figure 4H

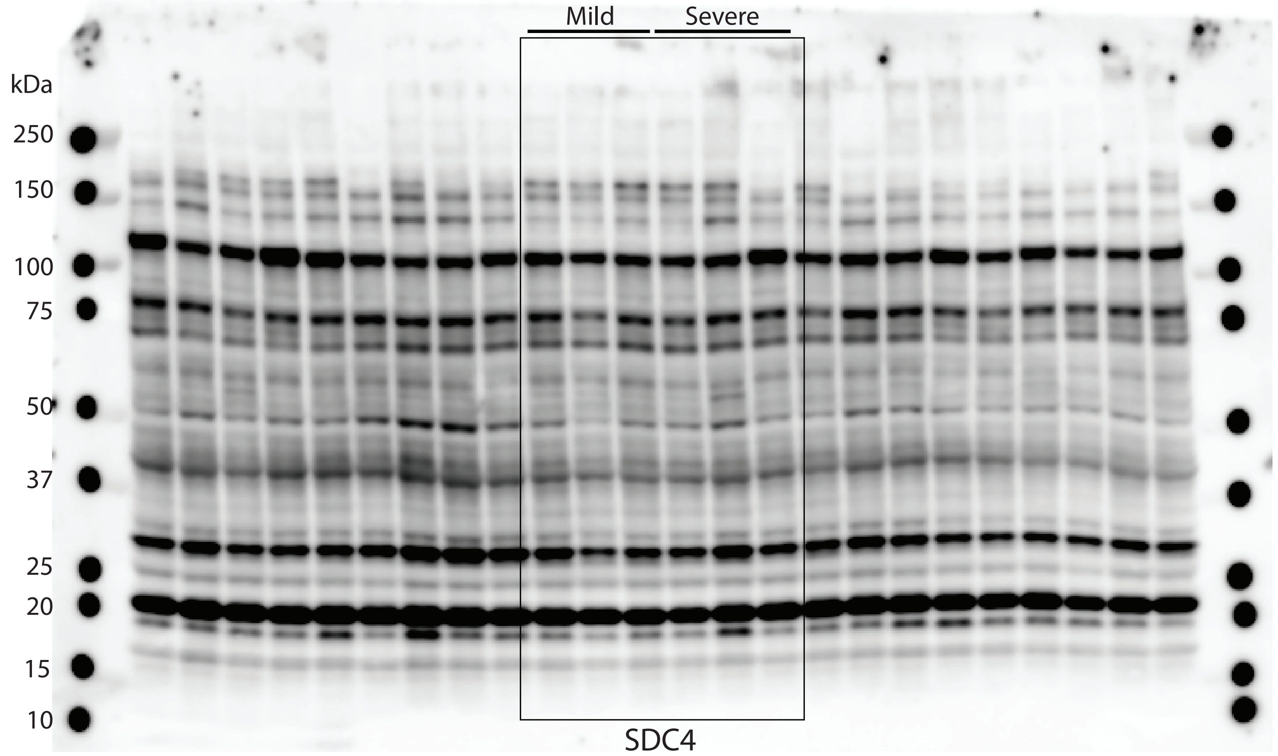

SDC4

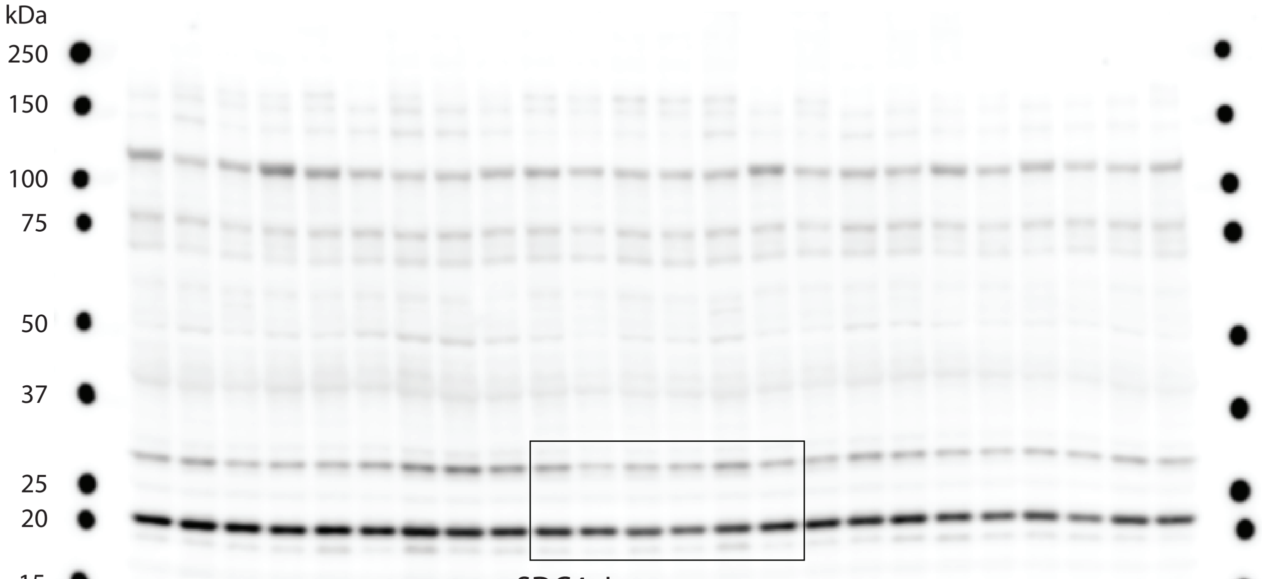

SDC4- Lower exposure

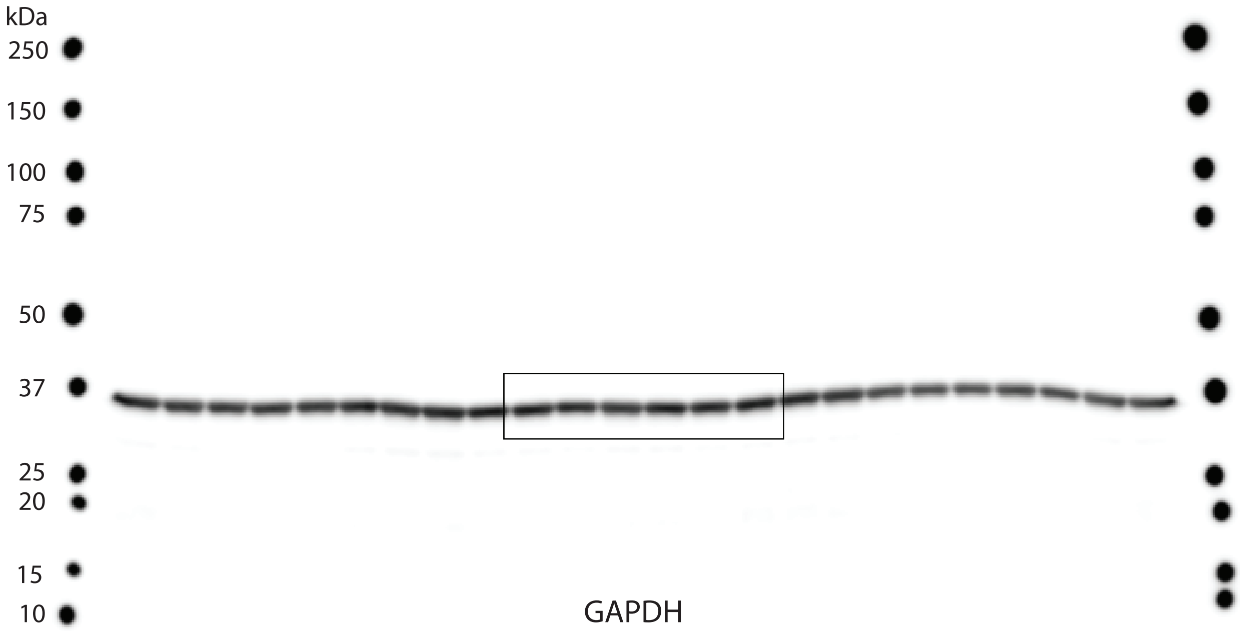

GAPDH

Full length blots for figure 5A

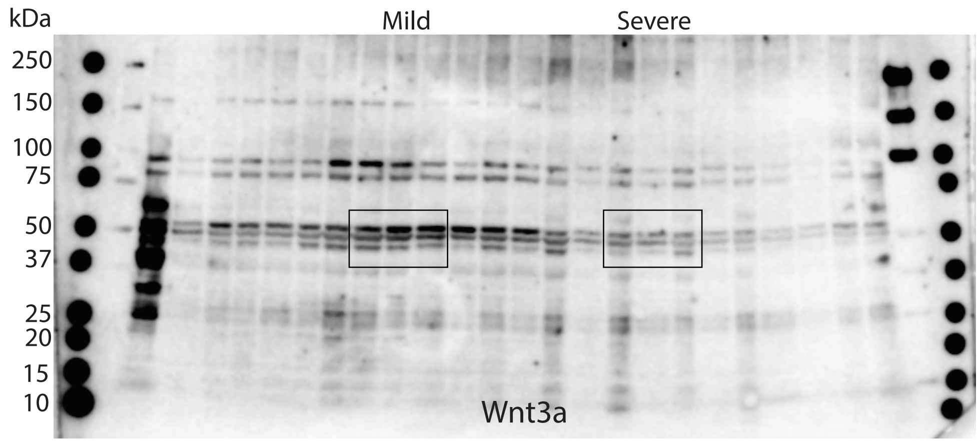

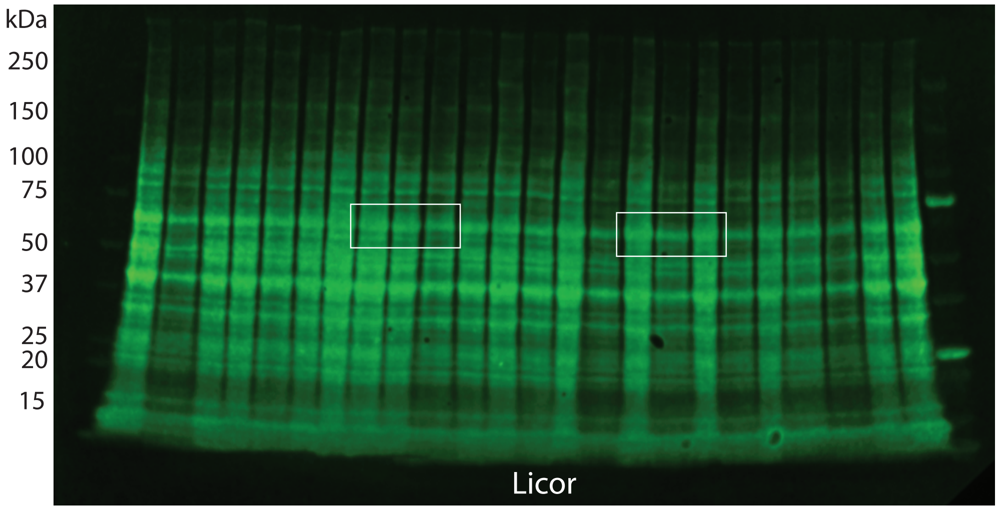

Full length blots for figure 5B

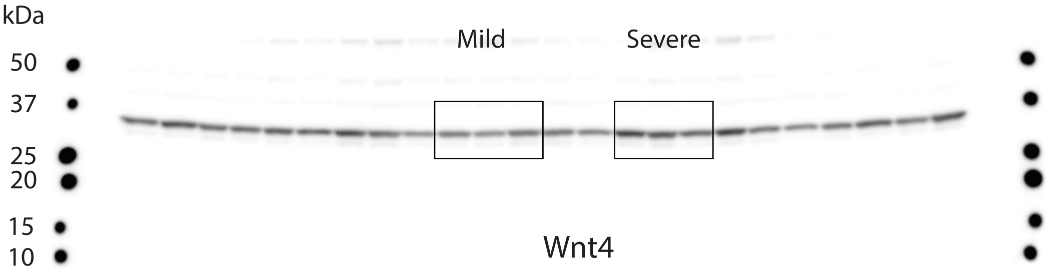

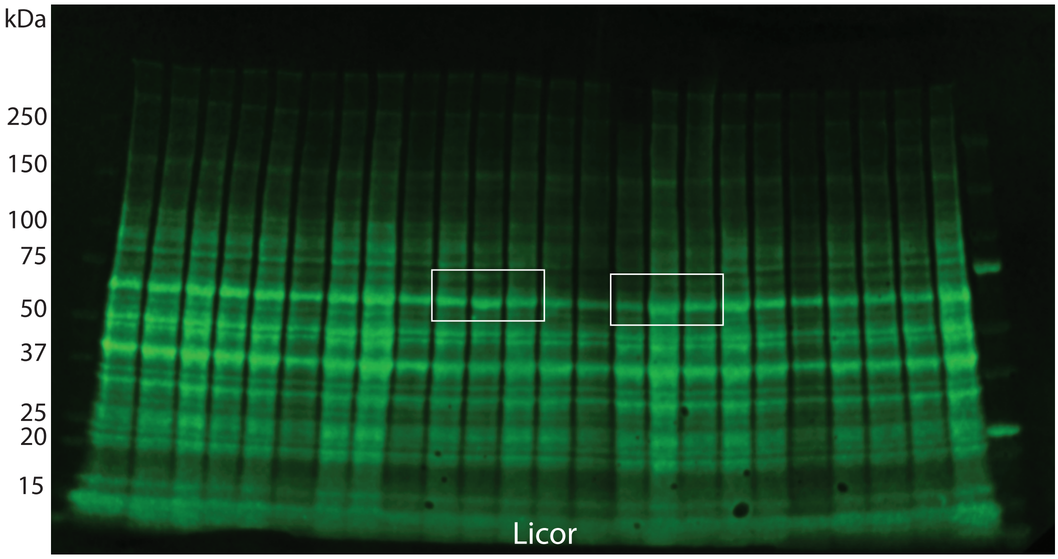

Full length blots for figure 5C

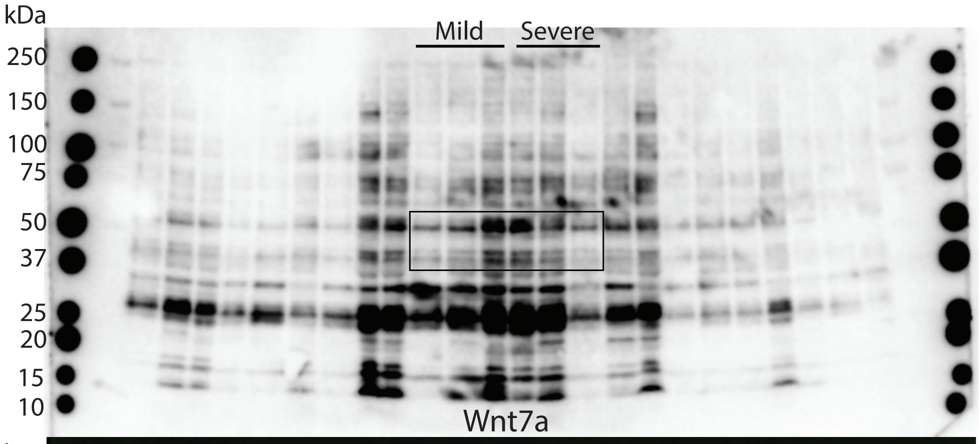

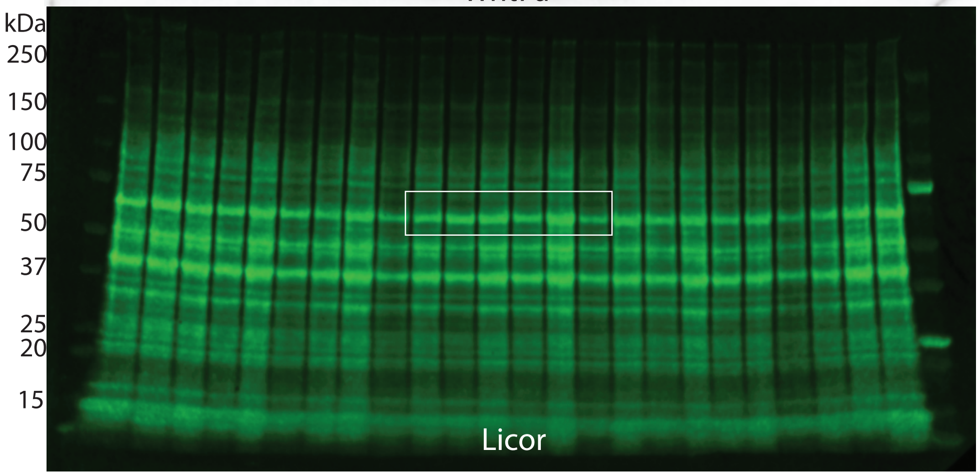

Full length blots for figure 5D

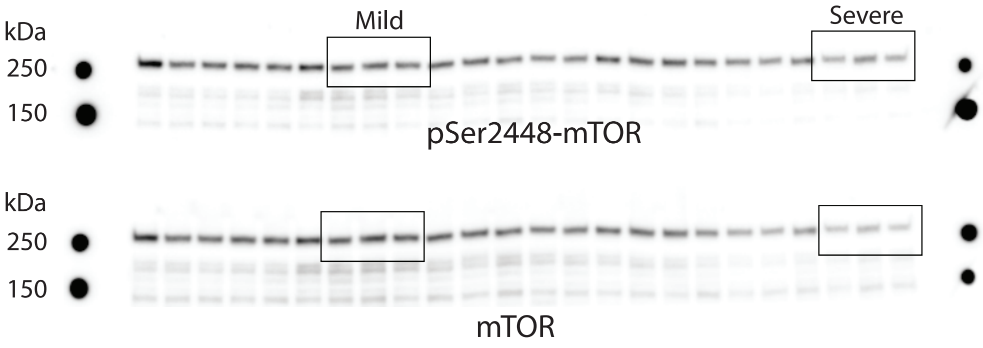

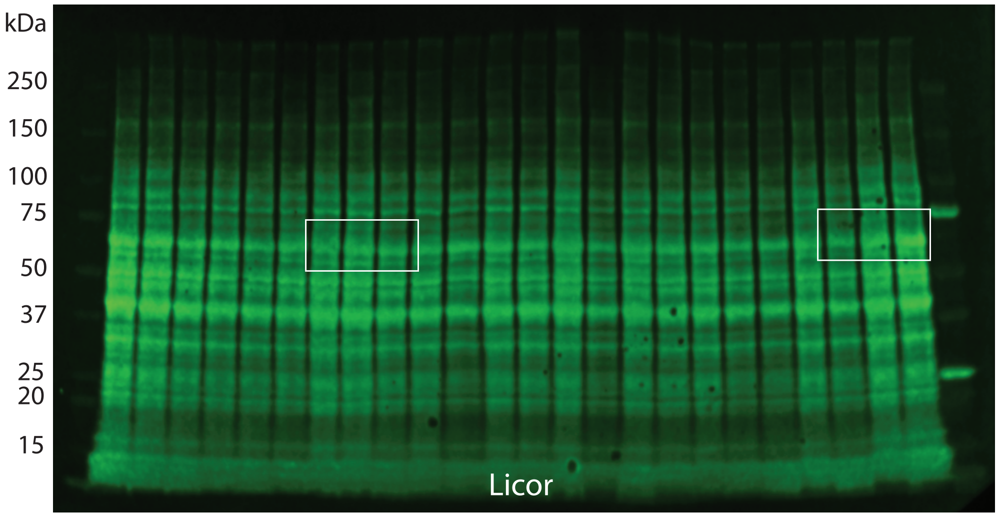

Full length blots for figure 5E

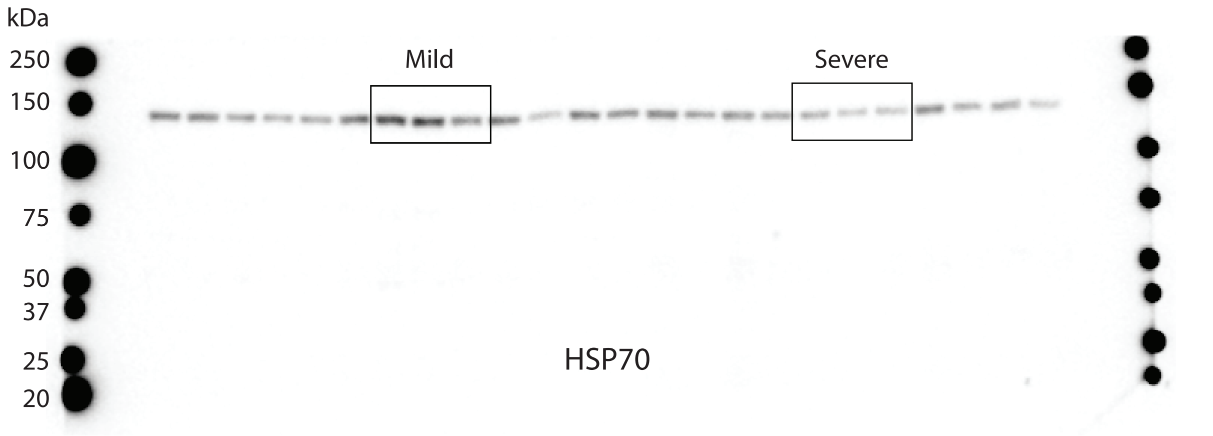

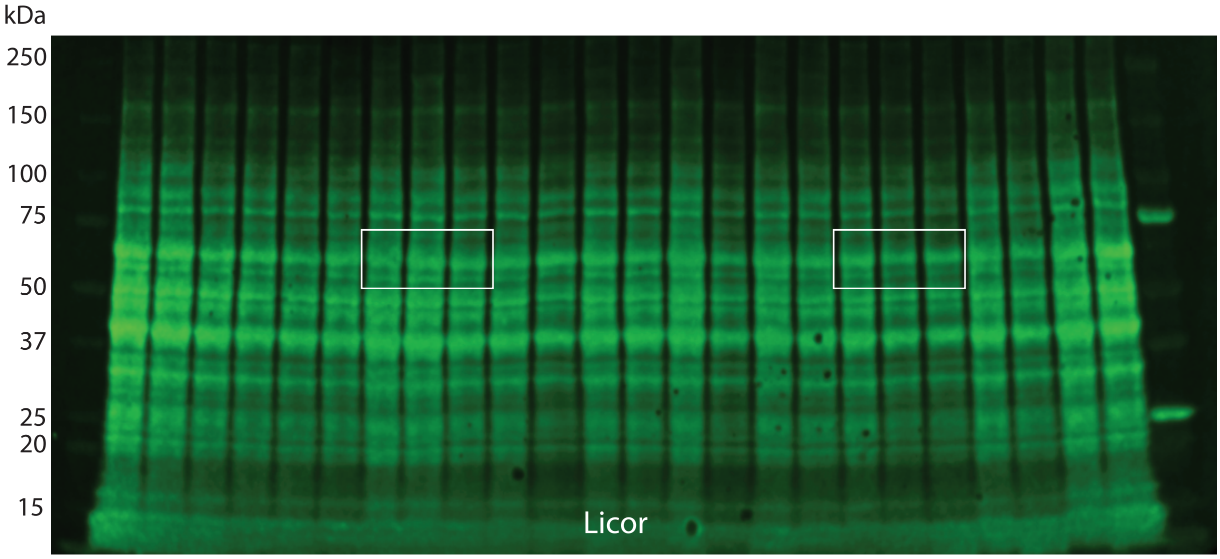

Full length blots for figure 5F

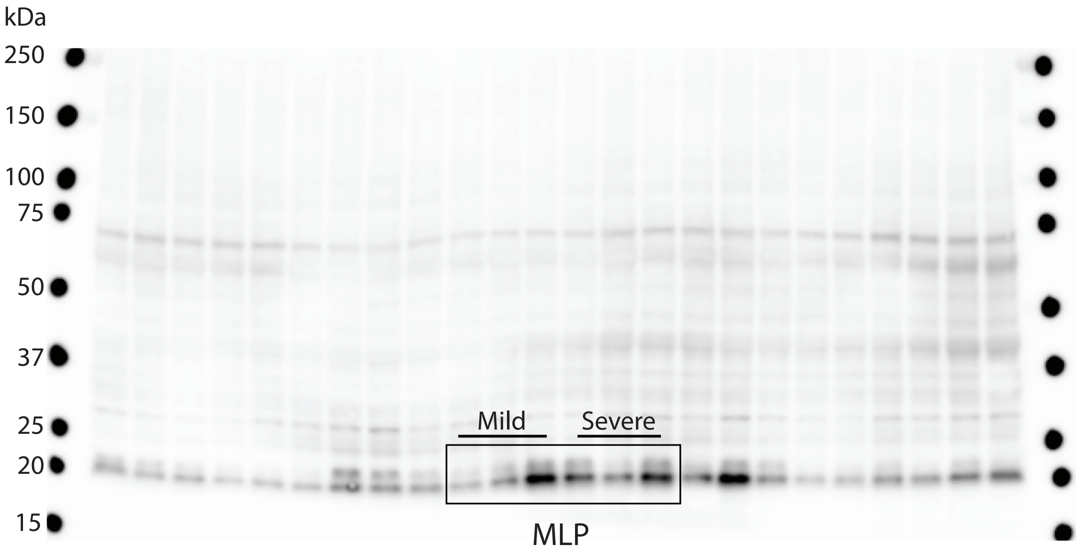

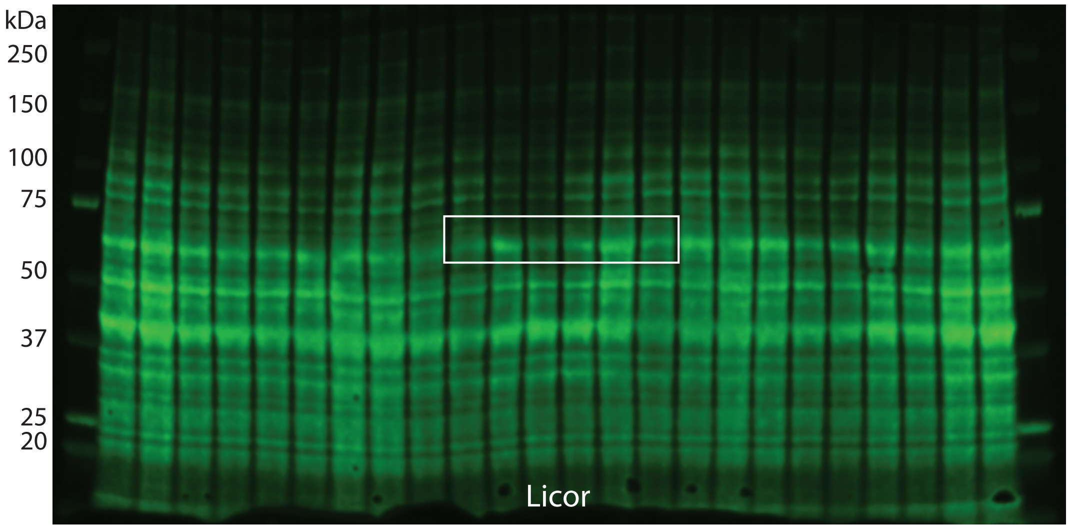

Full length blots for supplementary figure 1A

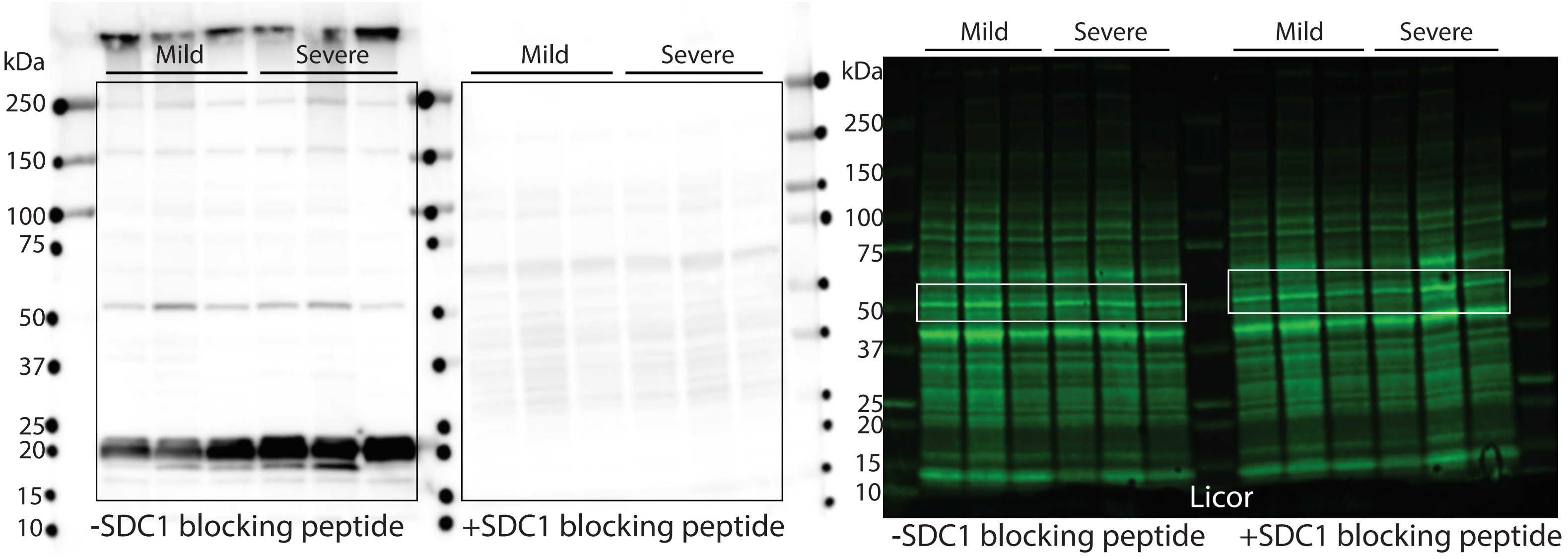

Full length blots for supplementary figure 1B

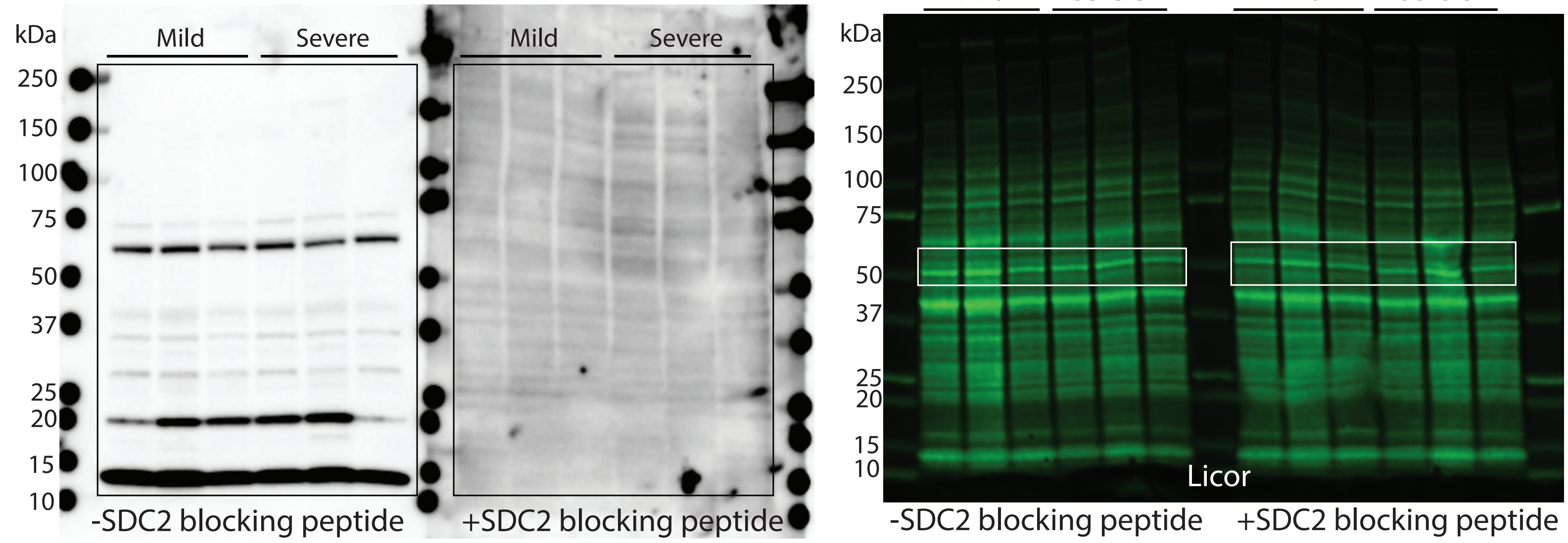

Full length blots for supplementary figure 1C

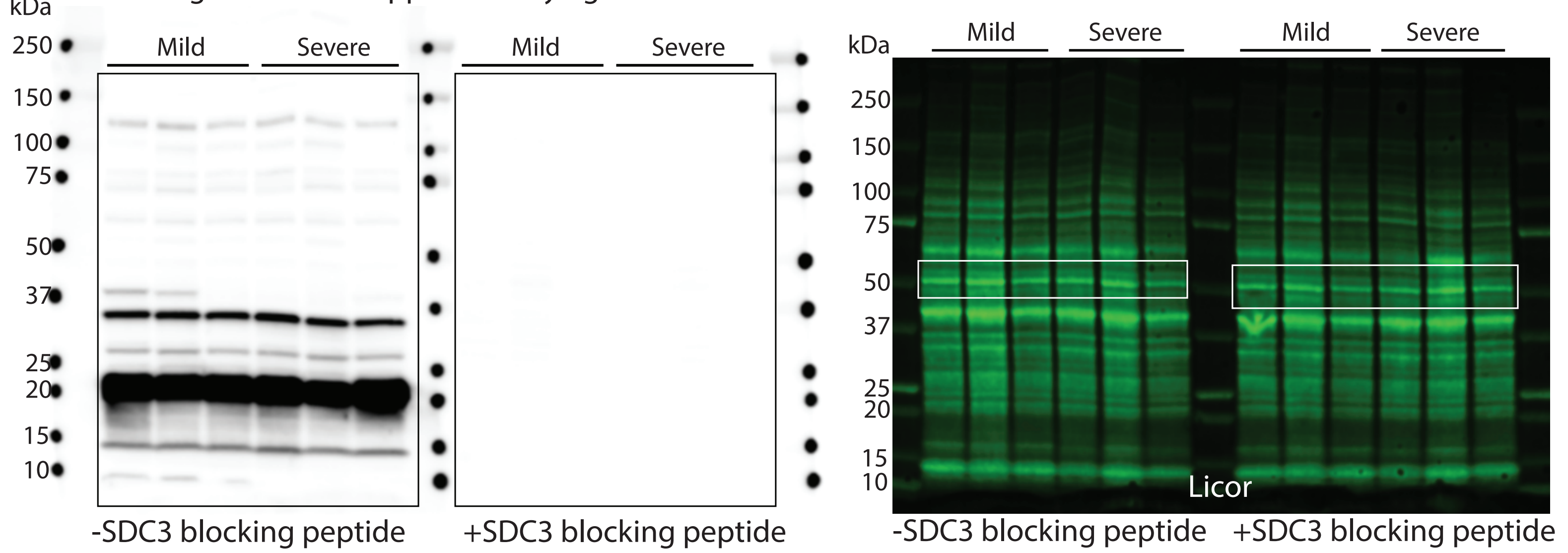

Full length blots for supplementary figure 1D

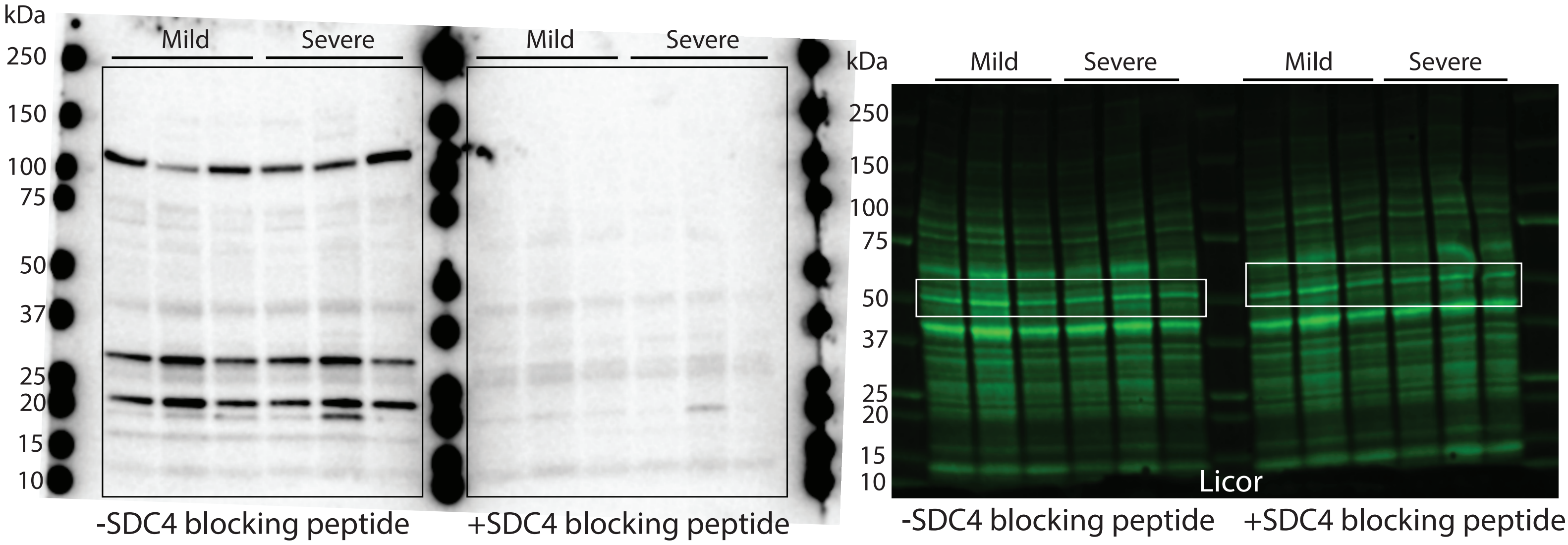

Full length blots for supplementary figure 2A

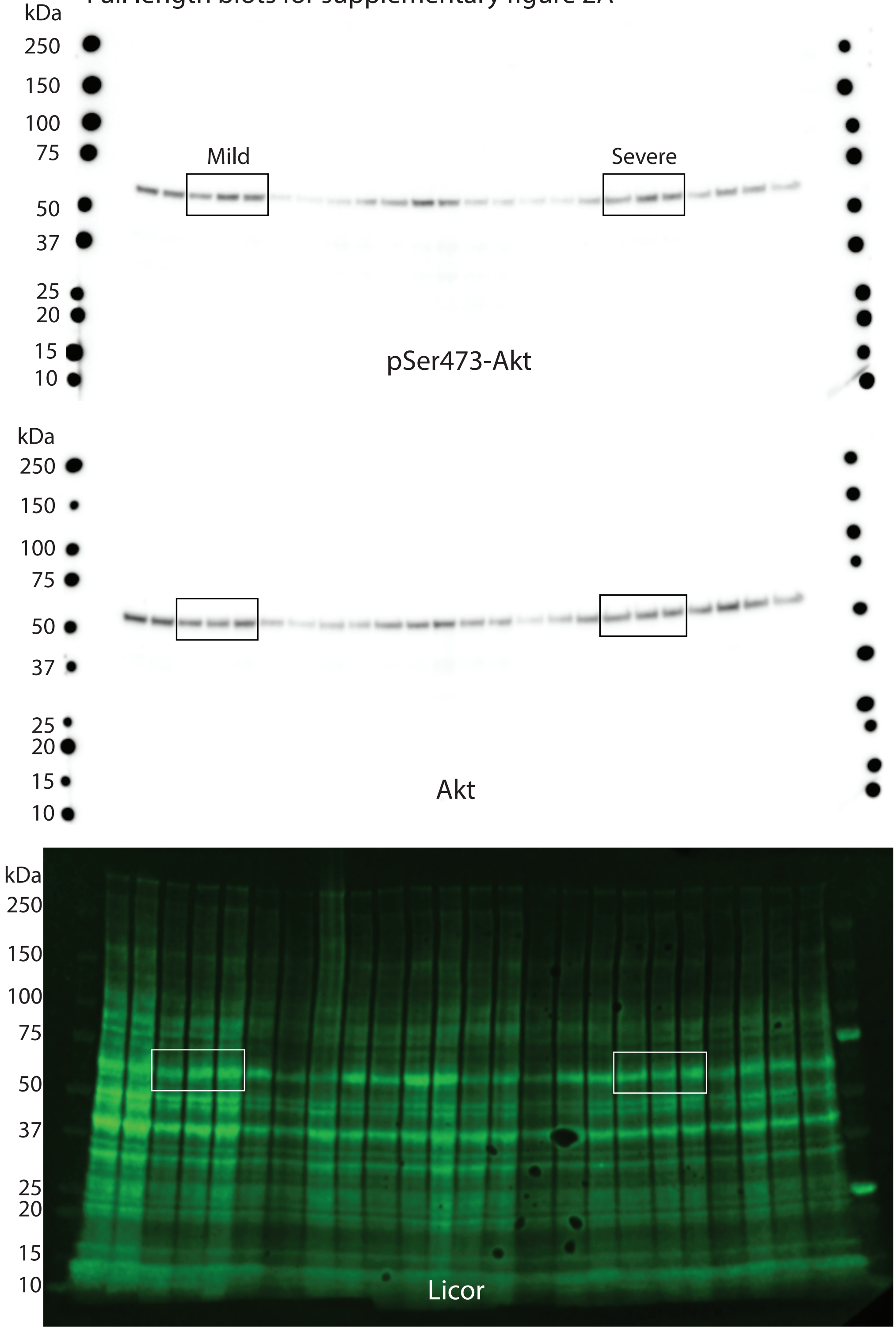

Full length blots for supplementary figure 2B

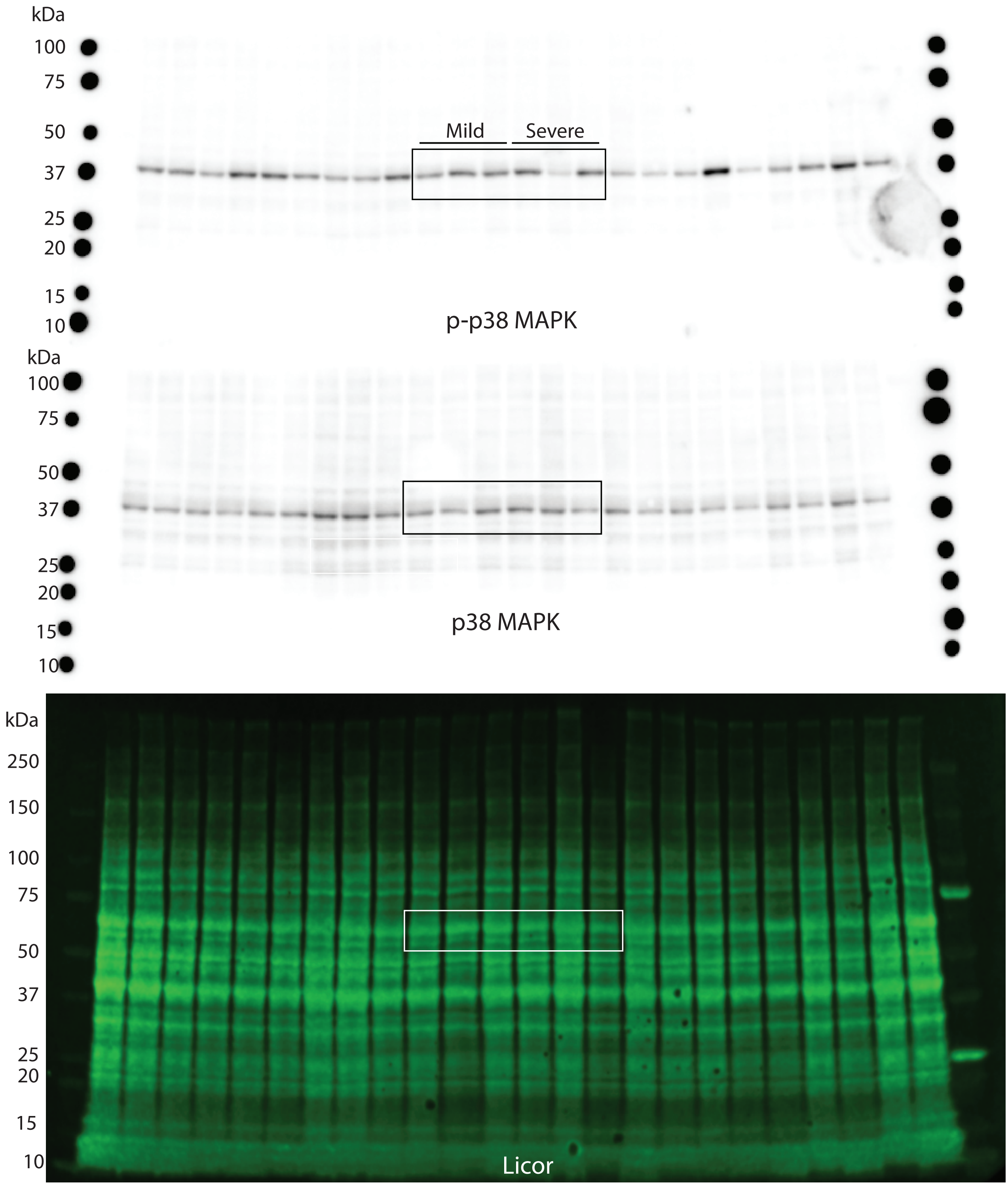

Full length blots for supplementary figure 2C

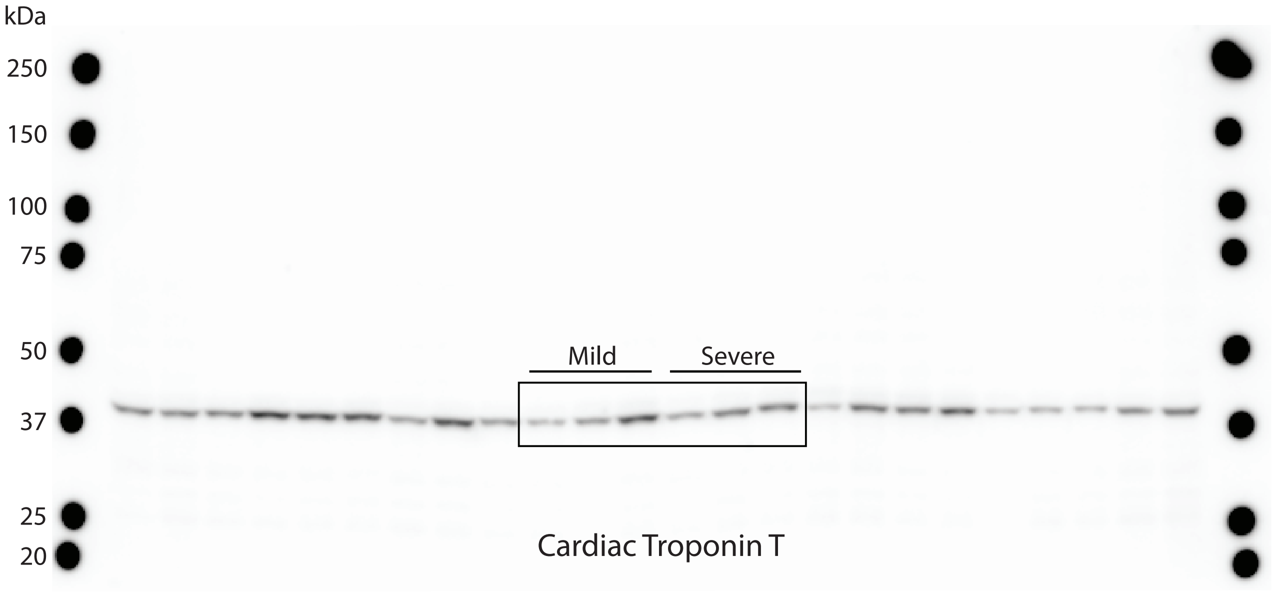

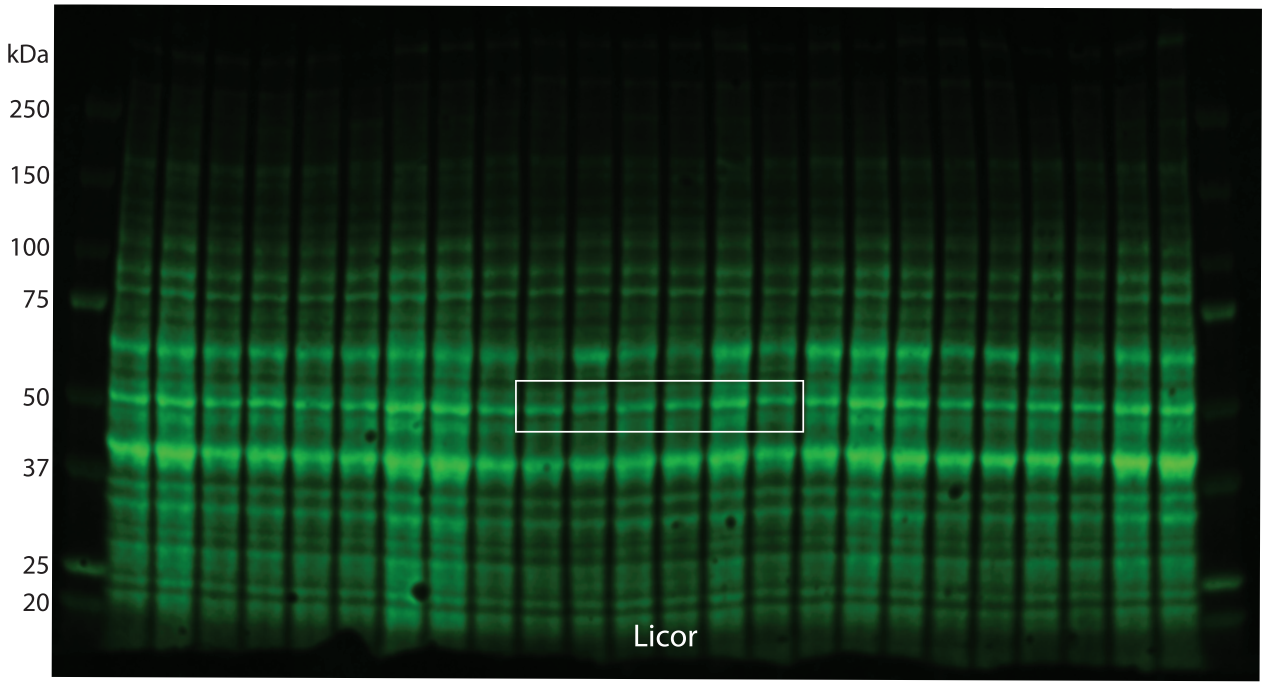

Supplement: Supplementary file 3 [file DataSheet1.pdf]
