# Supplementary material for: Cardiac implications of chicken wooden breast myopathy
Source: Front Physiol. 2025 Mar 5;16:1547661. doi: 10.3389/fphys.2025.1547661 (PMC11919848; doi:10.3389/fphys.2025.1547661)
Supplement: Supplementary file 4 [file Image1.pdf]

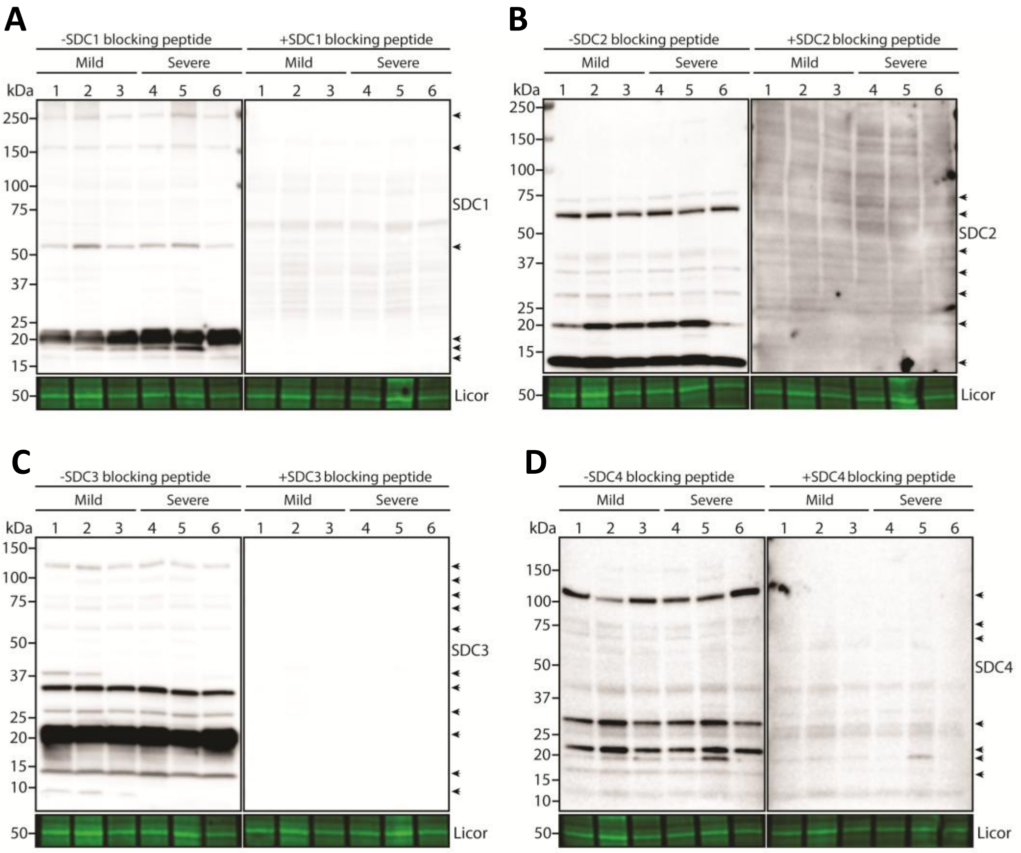

Supplementary figure 1. Epitope blocking experiments for syndecan 1-4. The specificity of (**A**) syndecan-1, (**B**) syndecan-2, (**C**) syndecan-3 and (**D**) syndecan-4 bands detected in chicken heart lysates (Fig. 4E-H) was analyzed using blocking peptides against the epitope of the respective antibodies. Membranes probed with the antibody and pre-incubated with the blocking peptide, are shown in the right panels, and membranes probed with the antibody only on the left. Syndecan positive bands are annotated with arrows on the right. Equal loading of duplicate samples was analyzed by licor staining (lower panels) (n=6).

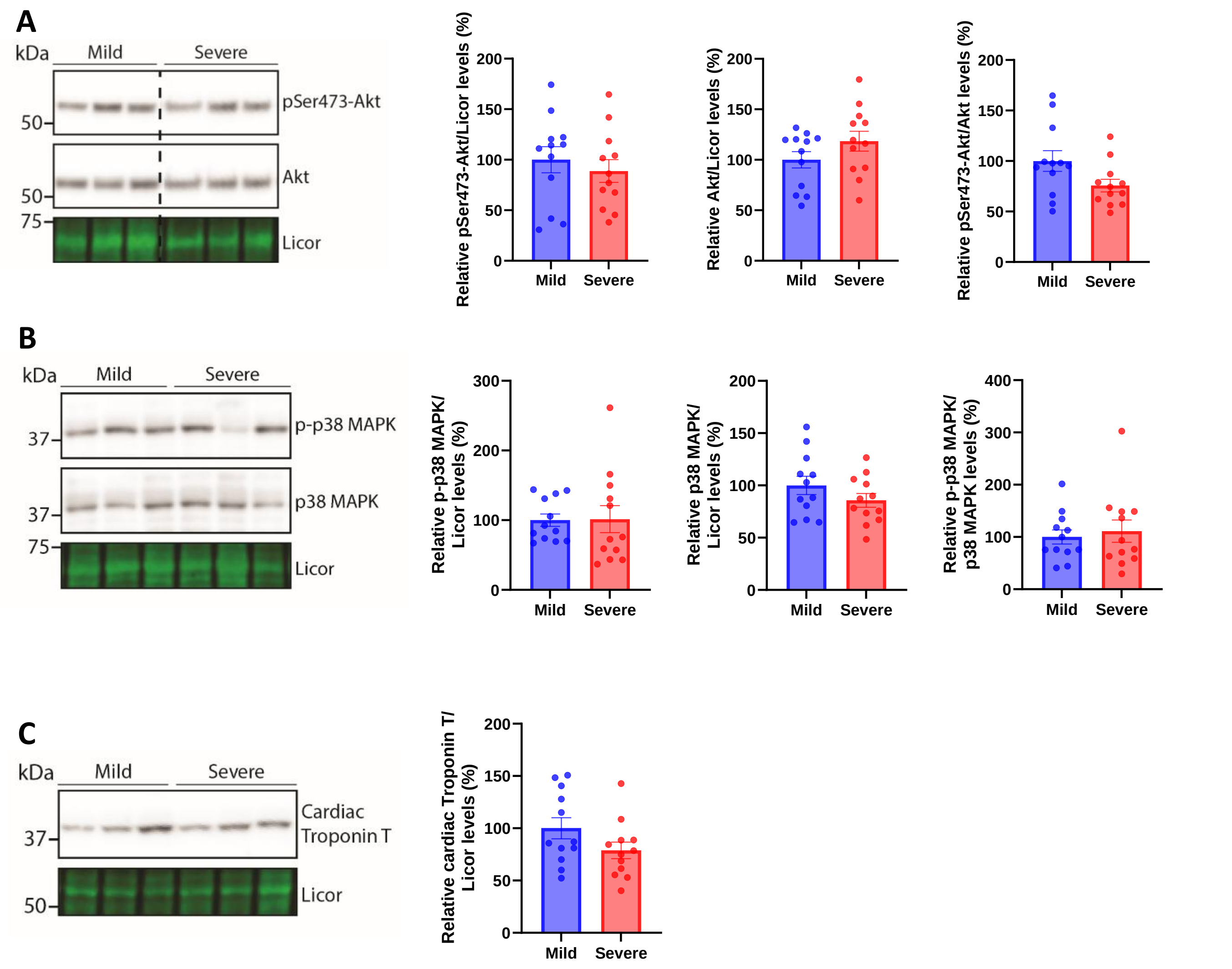

Supplementary figure 2. Akt and MAPK signaling, and cardiac troponin T were not altered in the hearts of WB-affected chickens. Immunoblotting of (**A**) pSer473-Akt and Akt, (**B**) pThr180/Tyr182-p38 MAPK and p38 MAPK, and (**C**) cardiac troponin T in hearts of mild and severely affected chicken (n=12). Licor was used to show equal loading (lower panels). Immunoblots are presented as mean ± SEM. Differences between groups were assessed with Mann-Whitney *U*-tests.

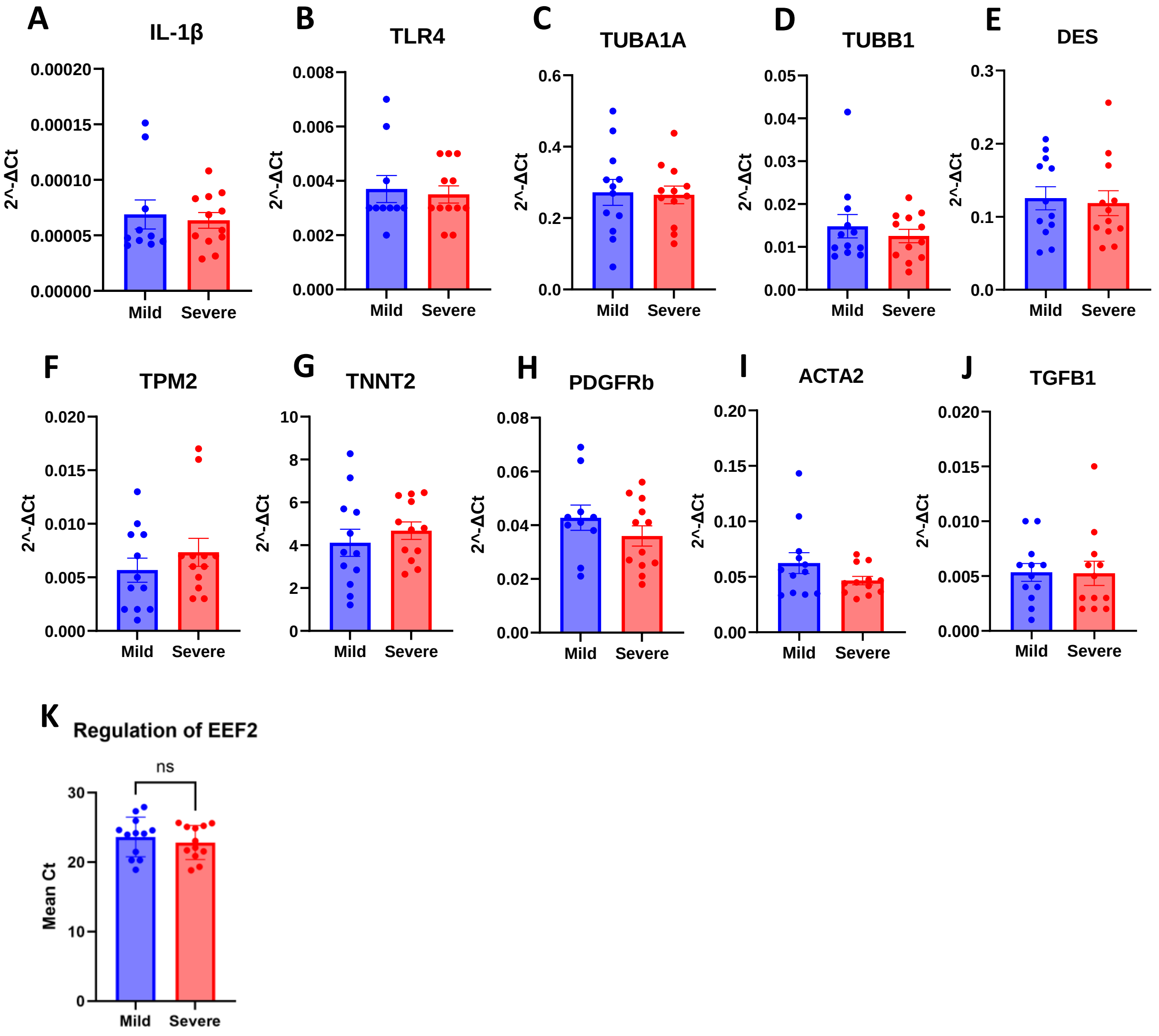

Supplementary figure 3. WB disease severity does not affect markers of inflammation, the cytoskeleton, myofibroblast, and fibrosis-triggering gene marker expression in the chicken heart. Gene expression by RT-qPCR of (**A**) IL-1β, (**B**) TLR4, (**C**) TUBA1A, (**D**) TUBB1, (**E**) DES, (**F**) TPM2, (**G**) TNNT2, (**H**) PDGFRb, (**I**) ACTA2, and (**J**) TGFB1 in mild and severely affected hearts (n=12). (**K**) The housekeeping gene EEF2 was not altered between groups. Data are presented as the fold change average relative to the mean of the mildly affected samples. Differences between groups were assessed with Welch`s t-tests.
